# MAP7 drives EMT and cisplatin resistance in ovarian cancer via wnt/β-catenin signaling

**DOI:** 10.1016/j.heliyon.2024.e30409

**Published:** 2024-04-29

**Authors:** Qingqing Chen, Shaojing Li, Furong Fu, Qunhuan Huang, Rong Zhang

**Affiliations:** a The Third School of Clinical Medicine,Southern Medical University, Guangzhou, 510500, China; b Shanghai Fengxian District Central Hospital, 6600 Nanfeng Road, Fengxian District, Shanghai, 201400, China; c Shanghai Municipal Hospital of Traditional Chinese Medicine, Shanghai University of Traditional Chinese Medicine, Shanghai, China; d Pingyang Hospital affiliated to Wenzhou Medical University, No.555, Kunao Road, Zhejiang Province, China

**Keywords:** MAP7, Wnt/β-catenin, EMT, Ovarian cancer, Cisplatin resistance, CBY1

## Abstract

**Methods:**

Our approach encompasses analyzing MAP7's expression levels across various datasets and clinical specimens, evaluating its association with patient outcomes, and probing its influence on ovarian cancer cell dynamics such as proliferation, migration, invasion, and apoptosis.

**Results:**

We have identified significant upregulation of MAP7 in ovarian cancer tissues, which correlates with advanced disease stages, higher pathological grades, and unfavorable prognoses. Functionally, the inhibition of MAP7 suppresses cancer cell proliferation, migration, and invasion while promoting apoptosis. Notably, the silencing of MAP7 attenuates the epithelial-mesenchymal transition (EMT) and disrupts Wnt/β-catenin pathway signaling—two critical processes implicated in metastasis and chemoresistance. In cisplatin-resistant A2780-DDP cells, the downregulation of MAP7 effectively reverses their resistance to cisplatin. Furthermore, the nuclear localization of MAP7 in these cells underscores its pivotal role in driving cisplatin resistance by modulating the transcriptional regulation and interaction dynamics of β-catenin.

**Conclusion:**

Our findings position MAP7 as a pivotal element in ovarian cancer advancement and cisplatin resistance, primarily through its modulation of EMT and the Wnt/β-catenin pathway. Its association with poor clinical outcomes underscores its potential as both a prognostic marker and a therapeutic target. Strategies aimed at MAP7 could represent a new frontier in combating chemotherapy resistance in ovarian cancer, emphasizing its significance in crafting complementary treatments for this disease.

## 1 Introduction

Ovarian cancer is among the leading threats to women's health globally, with its complex pathogenesis and challenging treatment options being central focuses of medical research [1]. The disease is particularly deadly due to its tendency to remain asymptomatic in early stages, leading to late diagnosis and limited treatment efficacy. According to the latest statistics from the World Health Organization (WHO) and the International Agency for Research on Cancer (IARC), over 300,000 women are diagnosed with ovarian cancer annually, resulting in approximately 200,000 deaths [2,3]. Despite efforts to improve early diagnosis and adjunct chemotherapy, the mortality rate of ovarian cancer has not seen significant improvement. The complexity of ovarian cancer's pathogenesis, which is still not fully understood, along with drug resistance, are major factors contributing to the poor prognosis of patients [4,5].

Microtubule-Associated Proteins (MAPs) are a family of proteins closely related to the dynamic equilibrium and stability regulation of microtubules [6,7]. By directly binding to microtubules, these proteins influence their polymerization and depolymerization, playing crucial roles in cell division, transport, signal transduction, and the maintenance of cellular architecture [8]. MAPs can be categorized based on their effects on microtubules, including polymer stabilizers (e.g., tau, MAP2, doublecortin), destabilizers (e.g., spastin, katanin), plus-end tracking proteins (e.g., EB1-3, XMAP215, CLASP), and motor proteins (e.g., dynein, kinesin). These seemingly unrelated proteins share a common feature: they contain microtubule-binding domains, and alterations in their protein expression levels appear to correlate with the aggressiveness of various human cancers and their sensitivity to microtubule-targeting chemotherapy drugs [[6], [7], [8], [9]].

MAP7, also known as Ensconsin, is a conserved non-motor MAP that participates in the regulation of microtubule organization, assembly dynamics, and stability [10]. It recruits kinesin-1 to microtubules, guiding the transportation of organelles and proteins from the nucleus to the cell periphery [11]. Recent studies have shown that MAP7 regulates cell migration capacity by modulating microtubule stability and reorganizing the cytoskeleton, affecting cell mobility. The stabilization of microtubules by MAP7 is vital for maintaining cell shape and ensuring accurate cell division [[11], [12], [13]]. During cell division, MAP7 is involved in controlling microtubule assembly and the precise separation of chromosomes [13]. MAP7 and its paralogue MAP7D1 regulate the migration and invasion of gastric cancer cells and the migration, adhesion, and cell cycle progression of cervical cancer cells through NF-κB and Wnt5α signaling [14]. Arlinda Dullovi et al. [15]found that MAP7 and MAP7D1 bind to various DNA repair proteins and participate in cell cycle regulation by modulating the recruitment of 53BP1 during the G1 phase of the DDR.

Our preliminary data analysis revealed that MAP7 is significantly overexpressed in ovarian cancer tissues compared to normal ovarian tissues. As one of the conserved non-motor MAPs, MAP7 is a protein closely associated with microtubule structure and dynamics regulation. It plays crucial roles in cell migration, division, and the establishment of cell polarity [16]. MAP7 interacts with microtubules and other cytoskeletal components, influencing various critical processes within the cell. MAP7 has been shown to affect the migration, cell cycle, and drug resistance of various tumor cells [15,17,18]. However, its role in ovarian cancer has not been reported, warranting further investigation.

## 2 Materials and methods

### 2.1 Cell lines and culture

Cancer cell lines (SKOV3, CAOV3, ES-2, A2780, OVCAR8, HEY, OVCAR429, and OVCAR3) were obtained from the Shanghai Institute of Cancer Research. All cells were cultured in various media: ES-2, CAOV3, A2780, and HEY in DMEM (HyClone Laboratories Inc., Logan, UT, USA); SKOV3, OVCAR8, and OVCAR429 in RPMI 1640 medium (HyClone Laboratories Inc., Logan, UT, USA). OVCAR3 cells were cultured in RPMI 1640 supplemented with 20 % (v/v) FBS (Gibco, New York, USA). All media were supplemented with 10 % (v/v) fetal bovine serum (FBS) and 1 % antibiotics (100 μg/mL streptomycin and 100U/ml penicillin). Cells were all maintained at 37 °C in a humidified incubator with 5 % CO_2_.

### 2.2 Cisplatin-resistant

Cell Culture A2780-DDP cells were developed by treating with 4 μM cisplatin pulses for 4 h (5 doses), followed by doses of 8 μM, 12 μM, 16 μM, and 20 μM, each for 4 h.

### 2.3 Bioinformatics analysis

Initially, two datasets were merged into one using the R package inSilicoMerging (version 2.10), and batch effects were removed following the approach by Johnson et al. [16] using the Limma (linear models for microarray data) method [19]in the R package limma (version 3.40.6), setting the threshold at Fold Change >1.2 and p < 0.05 to identify gene differences. Gene set enrichment analysis was performed with the R package clusterProfiler. The protein interaction network was constructed using the BIOGRID database (https://thebiogrid.org/), with key genes identified through Cytoscape (version 3.9.1), Gene expression profiling interactive analysis (GEPIA) (http://gepia2.cancer-pku.cn/#index) was used to display RNA sequencing expression in OC samples from the TCGA and GTEx projects. Kaplan-Meier plotter (https://kmplot.com/analysis/) assessed the impact of MAP7 on OC survival. p < 0.05 was considered statistically significant.

### 2.4 RNA-seq

Total RNAs isolated from CAOV3 transfected with siMAP7 or control siRNA by Trizol reagent following the manufacturer's instructions. RNA quality was assessed

using an Agilent Bioanalyzer 2100 (Agilent technologies, Santa Clara, CA, US) and sent for library preparation. Total RNA was amplifed, labeled, and purifed by RNAClean XP Kit (Beckman Coulter, Inc., Kraemer Boulevard Brea, CA, USA) and RNase-Free DNase Set (QIAGEN, GmBH, Germany) following the manufacturer's guidelines. Then, the purifed RNA was sequenced by Illumina novaseq 6000 platform by Majorbio Genomics (Shanghai, China), followed by analyzing the sequence data using GRCm38.p10 genome database.

### 2.5 Cell viability assay

Cells in logarithmic growth phase were seeded at 2000 cells/well in 96-well plates, incubated at 37 °C with 5 % CO_2_ overnight. After incubation (24h, 48h, 72h, and 96h), 100 μL of fresh medium containing 10 μL of CCK-8 solution (Shanghai TaoShu Biotechnology Co., Ltd., China) was added and incubated for 1.5 h at 37 °C. Absorbance was measured at 450 nm using a microplate reader (Bio-Rad, CA, USA).

### 2.6 Colony formation assay

Ovarian cancer cell lines were digested, suspended in serum-free medium, and reseeded in six-well plates at a density of 2000 cells/well with three biological replicates. After approximately 14 days of culture at 37 °C until visible colonies formed, cells were fixed with 4 % paraformaldehyde (Biying Tian Biotechnology, China, Shanghai) for 15 min and stained with 0.5 % crystal violet solution for 30 min. Colonies were photographed under an inverted microscope at 4 × magnification, and clone counts were conducted using Image J (a cluster is counted if it contains more than 50 cells).

### 2.7 Cell migration and invasion assays

Migration assays were performed using a 24-well Boyden chamber (8 μm pore size; Corning Costar, USA) with 1 × 10^5 cells in 200 μL serum-free medium loaded into the top chamber and 700 μL medium with 20 % FBS in the lower chamber. After 24 h of incubation, cells that migrated to the lower surface of the membrane were fixed with 4 % paraformaldehyde for 15 min and stained with 0.1 % crystal violet solution for 30 min. Photographs were taken using an inverted fluorescence microscope (Olympus Corporation, Japan), and cells in 5 fields of view were counted. BD Matrigel (BD Biosciences, USA) was thawed overnight at −4 °C to liquid before being diluted with serum-free medium at a 1:8 ratio for coating the upper chamber in invasion assays.

### 2.8 siRNA transfection

Cells were plated at 50–60 % confuence in 6 well cell culture plates. CAOV3 and OVCAR3 were transfected with siMAP7 or with control siRNA. The sequences of the siRNA used were as follows: si-MAP7-1, sense (5′-3′): AGCCCACATGGAGTCGCTTTACTC; si-MAP7-2, sense (5′-3′): CAGGCAGCTTAGGAACTAG and si-control, sense (5′-3′): TTCTCCGAACGTGTCAC GT. SiRNA oligos were purchased by Gene Pharma (Shanghai, China). Transfection steps were performed according to the reagent operation manual of Lipofectamine 3000 (Invitrogen, Thermo Fisher Scientific, CA, USA).

### 2.9 Plasmid transfection

Plasmid Transfection Lentivirus for overexpressing MAP7 and a control sequence were purchased from GenePharma (Shanghai, China). All these plasmids were

packaged into virus particles using HEK 293T cells and the viral titers were determined. Then the target cells were infected with 1 × 10^8^ lentivirus-transducing units with 6 μg/mL polybrene (Sigma-Aldrich, St. Louis, MO, USA). The

infected cells were then screened with 2 μg/mL puromycin after 72 h. The efciency of the overexpression was verifed.

### 2.10 Flow cytometry (FCM) for apoptosis

Detection Apoptosis was assessed using the Annexin V-FITC Apoptosis Detection Kit (Beyotime, Shanghai, China). Cells were collected, washed twice with PBS, and stained with Annexin V-FITC and PI in the dark at room temperature for 15 min. Apoptosis was evaluated using a FACS Calibur flow cytometer (BD Biosciences, San Jose, USA).

### 2.11 Real-Time quantitative PCR (qPCR)

Total RNA was extracted from cell lines using TRIzol (Invitrogen, Thermo Fisher Scientific, California, USA) according to the manufacturer's instructions. Reverse transcription was performed using a kit from Promega (Madison, Wisconsin, USA). Subsequently, qPCR was carried out using a 7300 Real-Time PCR System (Thermo Fisher Scientific, California, USA) with SYBR Green PCR Master Mix (Takara, Osaka, Japan). The primer sequences for MAP7 were as follows: forward, 5′-CTGCTAGGTGAGGGGAACTG-3′, and reverse, 5′-GGAGCGAGATCCCTCCAAAAT-3'. RPS18 was used as an internal control, and relative gene expression was analyzed using the 2^^−ΔCt^ method.

### 2.12 Patient information and clinical samples

Clinical information and samples (Table 1) were retrieved from the Department

**Table 1:** The relationship between the expression of MAP7 and the clinicopathological features of ovarian carcinoma.

Catalog	MAP7 high	MAP7 low	Total	X^2^	P
Classification					
Ovarian cyst	4(2.82 %)	138(97.18 %)	142	151.948	＜0.001***
Ovarian cancer	78(79.59 %)	20(20.41 %)	98		
Ege					
≤50y	22(59.46 %)	15(40.54 %)	37	5.025	0.025*
>50y	49(80.33 %)	12(19.67 %)	61		
Total	71(72.45 %)	27(27.55 %)	98		
Stage					
+II	27(55.1 %)	22(44.9 %)	49	16.962	＜0.001***
III + IV	45(91.84 %)	4(8.16 %)	49		
Total	72(73.47 %)	26(26.53 %)	98		
Grade					
High-grade serou OC	51(86.44 %)	8(13.56 %)	59	19.228	＜0.001***
Low-grade serous OC	11(40.74 %)	16(59.26 %)	27		
Total	62(72.09 %)	24(27.91 %)	86		

of Obstetrics and Gynecology, Fengxian District Central Hospital and the Department of Gynecology, Changzhou Maternal and Child Care Hospital, from 2004 to 2018. We included 98 cases of serous ovarian carcinoma and 142 cases of ovarian serous cystadenoma as controls. Paraffin-embedded samples from patients who underwent surgery were confirmed by professional gynecological pathologists.

### 2.13 Immunohistochemistry (IHC)

Analysis Tissue sections of 4 μm thickness were used for IHC staining. The antibody dilution for MAP7 rabbit polyclonal antibody (Merck, Germany) was 1:200, and for goat anti-rabbit IgG HRP antibody (Abcam, Inc., Massachusetts, USA) was 1:1,00. Following the manufacturer's recommendations, sections were incubated in xylene and a graded series of ethanol for dehydration and clarity. Endogenous peroxidase activity was blocked in 0.3 % hydrogen peroxide at 37 °C for 10 min, followed by washing with phosphate-buffered saline (PBS) for 3 min. After antigen retrieval in 10 mmol/l citrate solution (pH 6.0) by heating for 2 min, slides were incubated with the primary antibody against MAP7 overnight at 4 °C. Slides were then washed with PBS three times and incubated with the secondary antibody at room temperature for 1 h. Tissue sections were visualized using an enhanced HRP-DAB kit (Tiangen Biotech, Beijing, China) under a Zeiss microscope. After immunostaining, sections were counterstained with hematoxylin, dehydrated in graded ethanol, cleared in xylene, and mounted with neutral resin. Two pathologists, blinded to the clinical information, evaluated the immunoreactive score (Scoring was conducted according to the ratio and intensity of positive-staining cells. The staining extent was scored as: 0–5% scored 0; 6–35 % scored 1; 36–70 % scored 2; more than 70 % scored 3. The staining intensity was scored as: 0 (negative), 1 (weak), 2 (moderate) and 3 (strong). The immunoreactivity score (IRS) = extent score × intensity score, resulting in low (0–2) and high [[3], [4], [5], [6], [7], [8], [9]] values for each specimen.).

### 2.14 50 % Inhibitory concentration (IC50)

Measurement Cells (1 × 10⁴ cells/well) were plated in 96-well plates and incubated for 24 h before being treated with varying concentrations of cisplatin (0 μM–72.9 μM) for 72 h. After the treatment, cell viability was assessed using the CCK-8 assay to measure the OD value.

### 2.15 Nuclear and cytoplasmic protein extraction

Nuclear and cytoplasmic proteins were extracted using a Nuclear and Cytoplasmic Protein Extraction Kit (Thermo Fisher) according to the manufacturer's instructions. Cells were harvested and washed with cold PBS. Cell pellets were resuspended in reagent A containing a cocktail of protease inhibitors and incubated on ice for 10 min. Cold reagent B was added to the tube, followed by incubation on ice for 1 min. The samples were then centrifuged at 12,000 g for 10 min. The supernatant contained cytoplasmic proteins, while the pellet was resuspended in nuclear protein extraction reagent containing a cocktail of protease inhibitors and vortexed every 10 min for a total of 40 min. After centrifugation at 12,000 g for 10 min, the remaining supernatant was the nuclear extract.

### 2.16 Co-immunoprecipitation (Co-IP) assay

1 mg of total protein was diluted in 1 mL IP buffer containing protease inhibitors. Each sample was incubated with 10 μg of antibody (anti-MAP7, anti-β-catenin, or anti-CBY1) or control nonspecific, species-specific IgG. Samples were incubated with Protein A/G agarose beads (Beyotime, Shanghai, China) at room temperature for 2 h, followed by magnetic separation of the beads and washing three times with IP buffer. The beads were then resuspended in 40 μL 1 × electrophoresis sample buffer. After boiling for 5 min, the samples were subjected to Western blot analysis.

### 2.17 Immunofluorescence (IF)

Staining Ovarian cancer cells were fixed with 4 % paraformaldehyde for 30 min, permeabilized with 0.5 % Triton X-100 for 10 min, and blocked with goat serum. Subsequently, the cells were incubated overnight at 4 °C with anti-MAP7 antibody (diluted 1:300, HPA029712, Merck, Germany) and anti-CBY1 antibody (1:50, sc-393327, Santa Cruz Biotechnology, USA), and then labeled with Alexa Fluor-488-conjugated secondary antibody (1:400, Mouse, A0428, Beyotime, Shanghai, China) and Alexa Fluor-647-conjugated secondary antibody (1:400, Rabbit, A0468, Beyotime, Shanghai, China) at room temperature. Then, cell nuclei were stained with 4′,6-diamidino-2-phenylindole (DAPI) staining solution (ab104139; Abcam) at room temperature. Finally, cells were imaged using a fluorescence laser microscope (Olympus, Tokyo, Japan). Phalloidin (C2203S, Beyotime, Shanghai, China) diluted in PBS with 1 % BSA at a 1:150 ratio was used for staining according to the manufacturer's instructions.

### 2.18 Western Blot

Cells were lysed with 1x IP lysis buffer (Solarbio, Shanghai, China) supplemented with a protease inhibitor mixture (Solarbio, Shanghai, China). The total protein concentration was determined using a BCA protein assay kit (Beyotime, Shanghai, China). Cell lysates were mixed with 5x SDS sample buffer (Solarbio, Shanghai, China) and boiled for 10 min, then 20 μg of protein were loaded per gel lane. After electrophoresis, proteins were transferred to nitrocellulose membranes and blocked with 10 % non-fat milk supplemented with 0.1 % Tween. The membranes were incubated with primary antibodies against MAP7 (1:3000 dilution, HPA029712, Merck, Germany), p-β-catenin (Ser33) (1:1000, 28772-1-AP, Proteintech Group, Inc, USA), E-cadherin (1:1000, 20874-1-AP, Proteintech Group, Inc, USA), N-cadherin (1:1000, 22018-1-AP, Proteintech Group, Inc, USA), Vimentin (1:1000, 10366-1-AP, Proteintech Group, Inc, USA), Wnt3a (1:1000, 26744-1-AP, Proteintech Group, Inc, USA), Snai1 (1:1000, 13099-1-AP, Proteintech Group, Inc, USA), CBY1 (1:1000, 12239-1-AP, Proteintech Group, Inc, USA), mouse monoclonal antibody against Wnt10b (1:1000, 67210-1-Ig, Proteintech Group, Inc, USA), and rabbit monoclonal antibody against β-catenin (1:7000, ab32572, Abcam, Inc. Massachusetts, USA). GAPDH (1:10000 dilution, ab181602, Abcam, Inc. Massachusetts, USA) was used as a loading control and incubated overnight at 4 °C. After washing three times with PBS, membranes were incubated with HRP-conjugated secondary antibodies (goat anti-rabbit IgG and goat anti-mouse IgG, Beyotime, Shanghai, China) at room temperature for 1 h. The signal intensity was quantified using a protein blot imaging system (Tanon 5200, China). The relative gray value of the target protein to the control GAPDH was set to 1. The results represent three independent experiments.

### 2.19 Statistical analysis

Data are shown as means ± SD. Statistical analyses were done using SPSS 20.0 for Windows (IBM). Cumulative survival time was calculated by the Kaplan-Meier method and analyzed by the log-rank test. Correlation of MAP7expression with categorical clinical variables in patients with OC was evaluated by χ^2^ test or Fisher's exact test. The student's t-test or one-way ANOVA was used for comparison between groups. Graphs were created using GraphPad 9.0 software (GraphPad, La Jolla, USA) and Biorender (Biorender-JavaShuo). A p value of less than 0.05 was considered

statistically significant (*P < 0.05, **P < 0.01, ***P < 0.001).

## 3 Results

### 3.1 MAP7 expression is upregulated in ovarian cancer and associated with poor prognosis

**Fig. 1:**
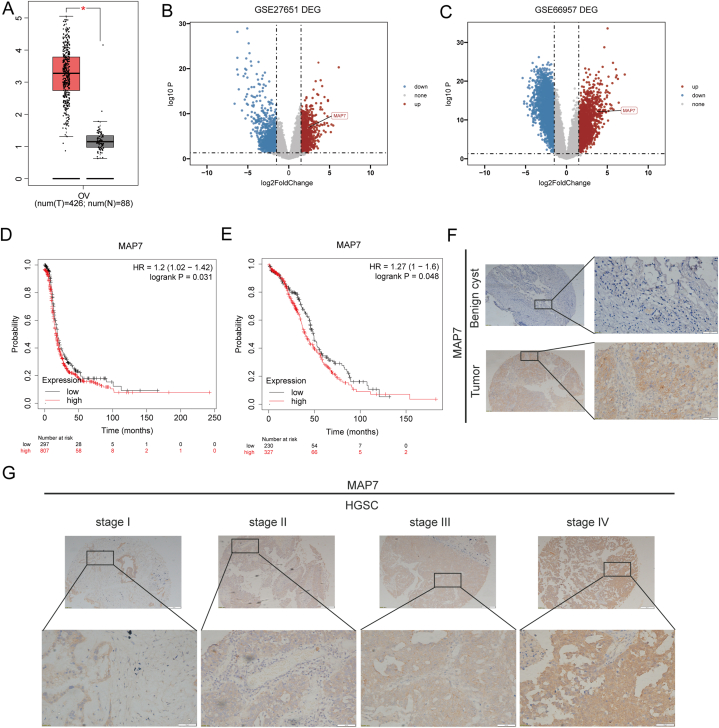
(A–G): High expression of MAP7 in ovarian cancer tissues. A. GEPIA data shows MAP7 levels in normal vs. cancerous ovarian tissues. (B–C). GSE27651 and GSE66957 analyses reveal higher MAP7 in ovarian cancer. D. KM-plot indicates worse PFS in high MAP7 expressers (p = 0.031). E. KM-plot links high MAP7 to lower OS (p = 0.048). (F–G). Immunohistochemistry demonstrates MAP7's presence across benign and malignant serous ovarian tumors and various FIGO stages.*p < 0.05 vs Control group.

Initially, our investigation commenced with an evaluation of gene expression profiles within GTEx and TCGA repositories, facilitated through the GEPIA platform. It was observed that ovarian cancer specimens exhibit markedly elevated mRNA levels of MAP7 compared to normal ovarian tissues (Fig. 1A). This upsurge in MAP7 expression was substantiated by analyses of two independent ovarian cancer patient cohorts (GSE27651 and GSE66957) employing R language (Fig. 1B and C). Prognostic implications were further elucidated using the Kaplan–Meier Plotter, revealing a significant linkage between heightened MAP7 expression and diminished progression-free survival (PFS) and overall survival (OS) among ovarian cancer patients (Fig. 1D and E), thus underscoring the potential of MAP7 upregulation as an indicator of poor clinical outcome.

Additionally, comparative assessments of MAP7 protein levels across 142 ovarian cyst and 98 ovarian cancer specimens indicated a pronounced increase in ovarian cancer tissues relative to ovarian cyst tissues (Fig. 1F), aligning with the mRNA expression trends observed in public datasets. A deeper dive into the association between MAP7 expression and clinicopathological traits among 98 ovarian cancer patients disclosed a significant correlation with patient age and FIGO stage (Fig. 1G), revealing elevated expression in older individuals and advanced disease stages (Table 1). Notably, among 86 serous ovarian cancer cases, MAP7 expression was predominantly higher in high-grade tumors, highlighting its potential role in the progression and aggressiveness of ovarian cancer.

### 3.2 Knocking down MAP7 inhibits the proliferation, migration, and invasion of ovarian cancer cells and promotes apoptosis

**Fig. 2:**
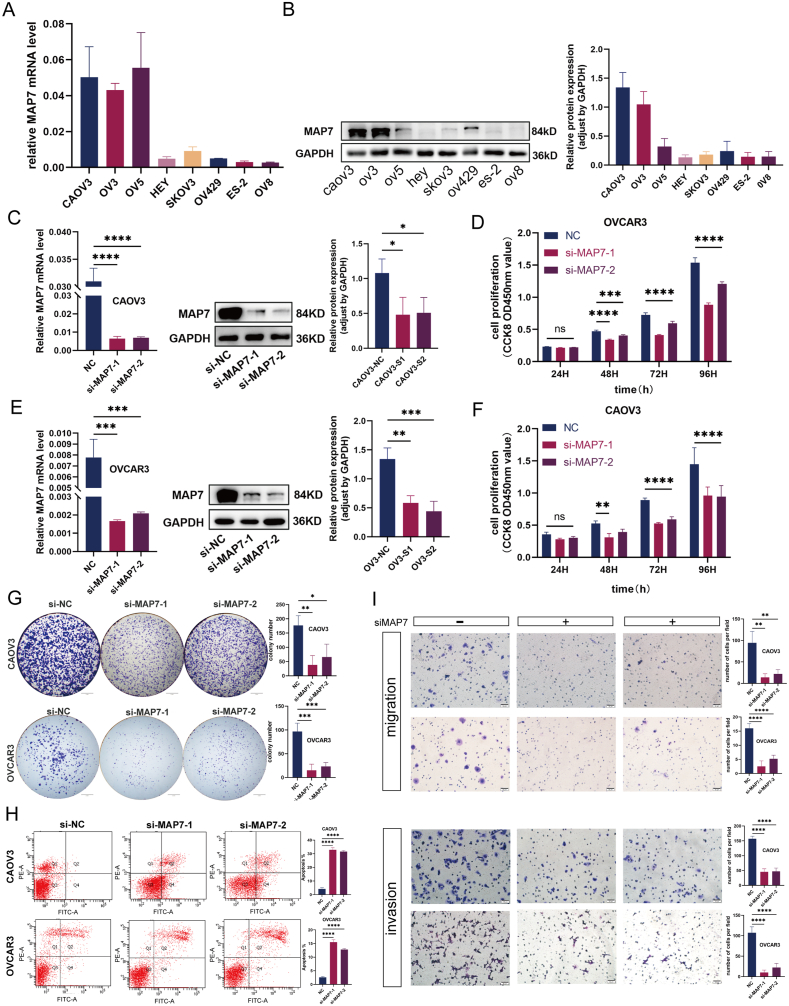
(A–I): Knockdown of MAP7 inhibits the progression of ovarian cancer cells. (A–B). MAP7 levels across cell lines. RPS18 as internal control for qPCR and GAPDH for Western blotting. (C, E). Knockdown efficiency confirmed. (D, F).Proliferation reduction shown by CCK-8. G. Decreased colony formation. H. Enhanced apoptosis via flow cytometry. I. Reduced migration and invasion in Transwell tests. Each experiment was performed in triplicate and independently repeated three times. (G: scale bar = 5 mm; I: scale bar = 50 μm) *p < 0.05 vs Control group, **p < 0.01vs Control group, ***p < 0.001 vs Control group and****p < 0.0001 vs Control group.

To elucidate MAP7's role in ovarian cancer, expression levels across different ovarian cancer cell lines were first quantified through Real-time qPCR and Western Blot analyses. Notably, MAP7 was predominantly expressed in CAOV3 and OVCAR3 cells, with comparatively lower levels observed in OVCAR8 and ES-2 lines (Fig. 2A and B). This differential expression guided the selection of CAOV3 and OVCAR3 for targeted MAP7 knockdown via si-RNA, successfully reducing MAP7 levels as confirmed through mRNA and protein assessments (Fig. 2C and E). Subsequent evaluations, utilizing CCK-8 proliferation assays and colony formation tests, illustrated a marked decline in ovarian cancer cell proliferation post-MAP7 knockdown, substantiating the gene's contributory role to cell growth (Fig. 2D–F, and G). Moreover, Transwell assays highlighted a significant reduction in the migratory and invasive capabilities of CAOV3 and OVCAR3 cells upon MAP7 inhibition (Fig. 2I). Complementary flow cytometry analysis further evidenced an increase in apoptosis rates within the MAP7 interfered cells compared to controls, underscoring MAP7's influence on cell survival mechanisms (Fig. 2H).

### 3.3 Overexpression of MAP7 enhances the proliferation, migration, and invasion of ovarian cancer cells while inhibiting apoptosis

**Fig. 3:**
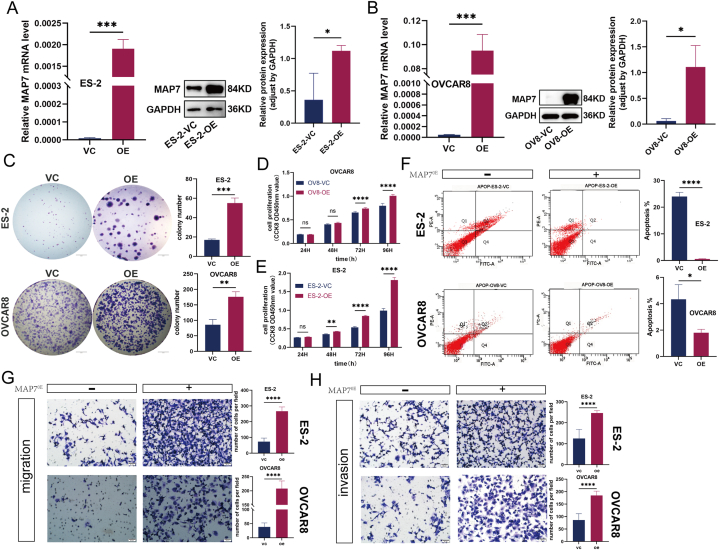
(A–H): Overexpression of MAP7 promotes the progression of ovarian cancer cells. (A–B). Confirmed MAP7 overexpression. RPS18 as internal control for qPCR and GAPDH for Western blotting. C. Increased colony formation shown. (D–E).Enhanced proliferation via CCK-8. F. Reduced apoptosis indicated by flow cytometry. (G–H). Greater migration and invasion in Transwell assays. Each experiment was performed in triplicate and independently repeated three times. (C: scale bar = 5 mm; G,H: scale bar = 50 μm) *p < 0.05 vs Control group, **p < 0.01vs Control group, ***p < 0.001 vs Control group and****p < 0.0001 vs Control group.

To substantiate MAP7's pivotal role in ovarian cancer cell, constructs overexpressing MAP7 were developed in ovarian cancer cell lines. Confirmatory Western blot and qRT-PCR analyses verified the enhanced expression of MAP7 (Fig. 3A and B). This genetic modification notably boosted both the proliferative and colony-formation capacities of ES-2 and OVCAR8 cells, underscoring MAP7's influence on cell growth and survival (Fig. 3C, D, E). Furthermore, Transwell assays revealed a pronounced increase in the migration and invasion capabilities of cells with MAP7 overexpression (Fig. 3G and H), while also significantly curtailing apoptosis (Fig. 3F). These comprehensive findings collectively affirm that MAP7 promotes the proliferation, migration, and invasion of ovarian cancer cells, concurrently impeding apoptotic processes.

### 3.4 MAP7 may promote epithelial-mesenchymal transition (EMT) in ovarian cancer cells by activating the wnt/β-catenin pathway

**Fig. 4:**
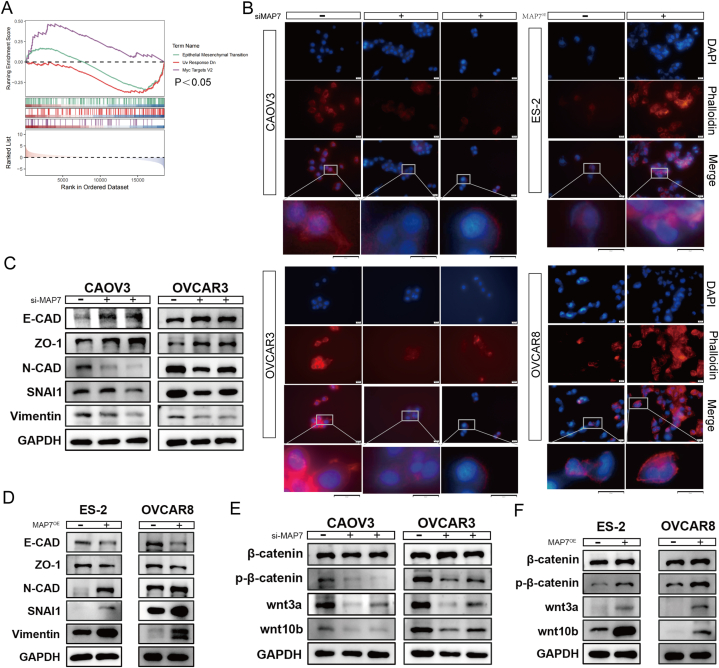
(A–F): MAP7 may promote epithelial-mesenchymal transition (EMT) in ovarian cancer cells by activating the Wnt/β-catenin pathway. A. MAP7 expression linked to EMT. B. MAP7's role in cytoskeletal organization shown by immunofluorescence. (C–D). MAP7's promotion of EMT. (E–F). Activation of the Wnt/β-catenin pathway by MAP7. Each experiment was performed in triplicate and independently repeated three times. (B: scale bar = 20 μm).

To decipher MAP7's mechanistic influence in ovarian cancer, we employed GSEA enrichment analysis on ovarian cancer samples from the TCGA database (n = 594), categorizing them based on MAP7 expression levels. The analysis indicated a correlation between MAP7 expression and epithelial-mesenchymal transition (EMT) processes (Fig. 4A). Given MAP7's role in stabilizing microtubules and remodeling the cytoskeleton, we hypothesized it might modulate EMT through these pathways. Phalloidin staining for F-actin was utilized to visualize MAP7-induced alterations in the actin cytoskeleton. The experiments revealed that MAP7 interference markedly diminished F-actin fluorescence intensity, reduced the number of filamentous bundles, and led to a more disorganized cytoskeletal structure in CAOV3 and OVCAR3 cells. Conversely, MAP7 overexpression in ES-2 and OVCAR8 cells enhanced F-actin fluorescence, resulting in denser and more numerous filamentous bundles, indicating a more structured cytoskeletal arrangement (Fig. 4B). These findings underscore MAP7's capacity to orchestrate cytoskeletal assembly, further implicating it in the regulation of EMT in ovarian cancer.

To elucidate MAP7's role in EMT within ovarian cancer, we analyzed EMT marker protein expression via Western blot. Post-MAP7 interference, CAOV3 and OVCAR3 cells exhibited increased levels of epithelial markers E-cadherin and ZO-1, and decreased mesenchymal markers N-cadherin, snai1, and vimentin (Fig. 4C). The reverse was observed in MAP7-overexpressing ES-2 and OVCAR8 cells, indicating MAP7's influence on EMT marker expression (Fig. 4D).

Considering the critical role of extracellular signaling and the Wnt/β-catenin pathway in driving EMT, where β-catenin acts both as an effector of Wnt signaling and as a cell adhesion component, we investigated the impact of MAP7 on this signaling pathway. Western blot analyses following MAP7 modulation showed changes in key Wnt/β-catenin pathway molecules. Knockdown of MAP7 led to reduced levels of wnt3a, wnt10b, and phosphorylated β-catenin in CAOV3 and OVCAR3 cells, indicating a dampening of pathway activity. Conversely, MAP7 overexpression in ES-2 and OVCAR8 cells resulted in increased expression of these components (Fig. 4E and F), pointing to MAP7's role in activating the Wnt/β-catenin pathway and thereby promoting EMT in ovarian cancer cells.

### 3.5 The expression of MAP7 is associated with platinum drug resistance in ovarian cancer cells

**Fig. 5:**
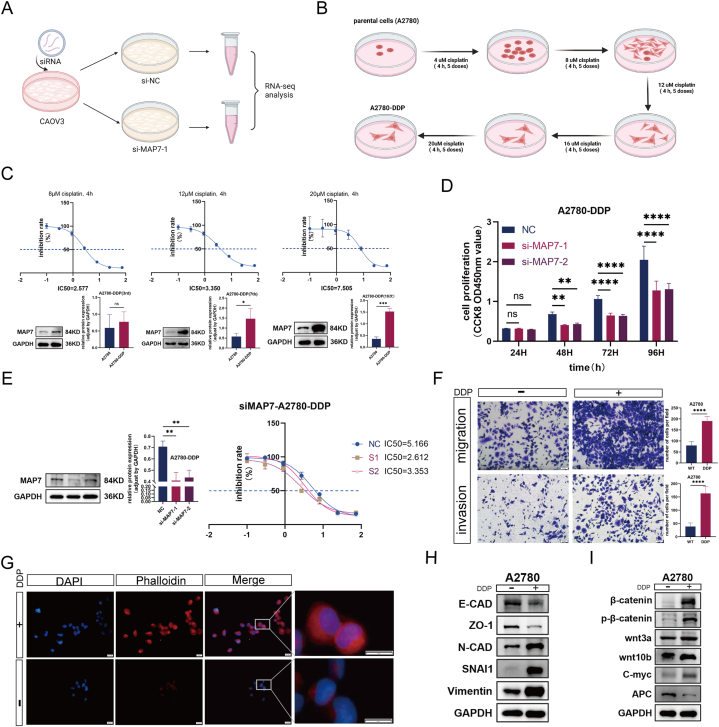
(A–K): The expression of MAP7 is associated with cisplatin resistance in ovarian cancer cells. A. RNA-seq submission schematic. B. A2780-DDP cell line development flowchart. C. Rising MAP7 levels with increased resistance. D. CCK-8 shows reduced A2780-DDP proliferation post-MAP7 knockdown. E. Lowered IC50 reflects decreased resistance. F. Enhanced A2780-DDP migration and invasion in Transwell assays. G. Cytoskeletal changes via immunofluorescence. H. Western blots reveal EMT activation. I. Wnt/β-catenin/C-myc pathway upregulation drives EMT and resistance. Each experiment was performed in triplicate and independently repeated three times. (H: scale bar = 50 μm; I: scale bar = 20 μm) ns, not significant, *p < 0.05 vs Control group, **p < 0.01vs Control group, ***p < 0.001 vs Control group and****p < 0.0001 vs Control group.

After 48 h of siRNA-mediated MAP7 knockdown, CAOV3 cells and control samples were sent to Majorbio Pharmaceutical Technology Co., Ltd., Shanghai, for quality control and RNA integrity analysis (Fig. 5A), followed by library preparation and sequencing. KEGG enrichment analysis linked MAP7 knockdown to platinum drug resistance in ovarian cancer cells (Supplemental Material 1,2).

In ovarian cancer research, in vitro cell line models have become effective tools for understanding the molecular mechanisms behind acquired chemotherapy resistance. To generate a cisplatin-resistant OC cell line (CisR OC), we exposed the OC cell line A2780 to cisplatin in a pulse dosing and gradually increasing dosage manner over eight months (Fig. 5B). These A2780-DDP cells were confirmed to have a cisplatin IC50 value (percentage inhibition at 72 h post-cisplatin treatment) that is at least 5–7 times higher than that of their parent cells (around 1uM for A2780). A2780-DDP cells showed changes in cell size, occasionally larger multinucleated "giant" cells, prominent nucleoli, increased nuclear pleomorphism, and increased numbers of cellular protrusions (dendrites). A2780-DDP also displayed stronger adherence to culture dishes compared to parental cells. Western Blot analysis showed MAP7 levels increasing with resistance development (Fig. 5C). Knocking down MAP7 in A2780-DDP cells significantly reduced their cisplatin resistance and proliferative ability (Fig. 5D and E).

Transwell assays revealed A2780-DDP cells had enhanced migration and invasion capabilities (Fig. 5F), and cytoskeletal reorganization was observed (Fig. 5G). Western blot analysis showed a shift towards an EMT phenotype in A2780-DDP cells with upregulation of mesenchymal markers and downregulation of epithelial markers (Fig. 5H). Western blot analysis targeted key molecules in the Wnt/β-catenin/C-myc pathway, including wnt3a, wnt10b, β-catenin, C-myc, and p-β-catenin. Findings revealed upregulation of these components in A2780-DDP cells relative to parental cells (Fig. 5I), indicating activation of the Wnt/β-catenin/C-myc pathway. This activation is implicated in promoting EMT and enhancing cisplatin resistance in A2780-DDP cells.

### 3.6 CBY1 may serve as a target for the action of MAP7 within the nucleus of A2780-DDP cells

**Fig. 6:**
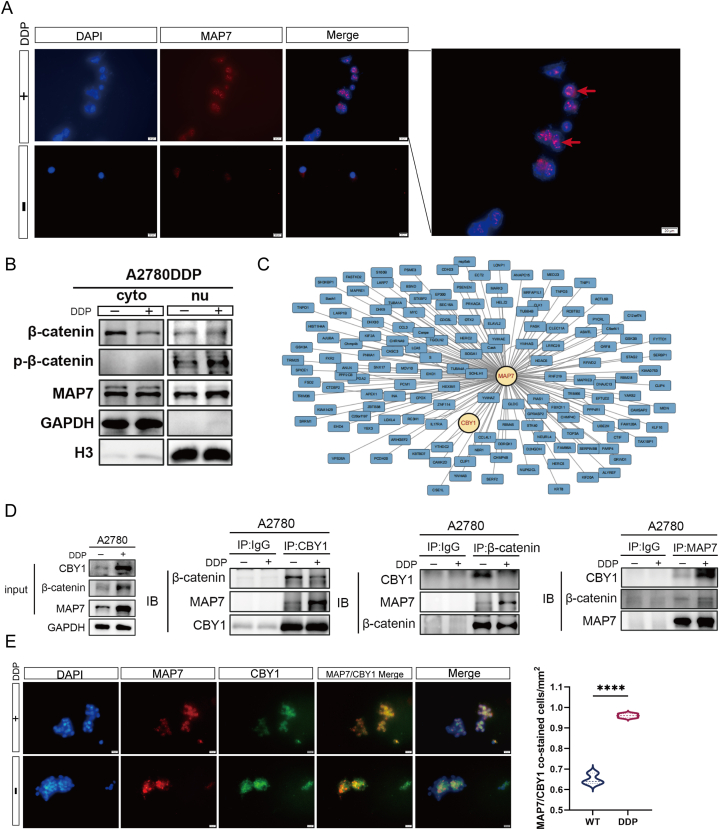
(A–D): CBY1 may serve as a target for the action of MAP7 within the nucleus of A2780-DDP cells. A: Elevated nuclear MAP7 in A2780-DDP. The white arrow shows the expression of MAP7 in the nucleus. B: Western blot highlights nuclear MAP7 and β-catenin translocation, GAPDH was used as a positive reference for cytoplasmic localization and H3 was used as a positive reference for nuclear localization. C: BioGRID database shows MAP7-CBY1 interaction. D: COIP reveals interactions between MAP7, CBY1, β-catenin. E. Immunofluorescence staining showing the co-localization of MAP7 and CBY1. Each experiment was performed in triplicate and independently repeated three times. (A, E: scale bar = 20 μm) ****p < 0.0001 vs Control group.

Immunofluorescence experiments revealed heightened MAP7 protein expression in ovarian cancer A2780-DDP cells, predominantly localized to the nucleus (Fig. 6A), indicating nuclear MAP7's key role in cisplatin resistance. Further analysis through Western blot of nuclear and cytoplasmic proteins highlighted an increase in nuclear MAP7 alongside reduced cytoplasmic β-catenin and elevated nuclear p-β-catenin (Fig. 6B). This shift, coupled with APC's significant downregulation in A2780-DDP cells (Fig. 5K), prevents effective β-catenin degradation, leading to its nuclear accumulation and suggesting MAP7's involvement in activating the Wnt/β-catenin/C-myc pathway, contributing to EMT and enhanced cisplatin resistance.

The spatial dynamics of MAP7, shifting between the nucleus and cytoplasm, imply its regulatory role in nuclear transport processes, potentially influencing the nucleocytoplasmic shuttling of RNA and proteins. MAP7 might interact with nuclear pore complexes or transport proteins, affecting the movement of molecules across the nuclear envelope. The specific functions of MAP7 within the nucleus, particularly its link to cisplatin resistance, remain an underexplored territory in current research. To identify nuclear targets of MAP7 and understand its role in transcriptional regulation and cellular function, we turned to large-scale mass spectrometry data from the BioGRID database, employing Cytoscape in R for protein-protein interaction (PPI) network visualization (Fig. 6C). Our focus centered on CBY1, a key inhibitor of nuclear Wnt/β-catenin signaling. CBY1's interaction with β-catenin limits its nuclear entry, dampening β-catenin's nuclear activities and consequent Wnt pathway activation [20,21]. A reduction in CBY1 could result in β-catenin hyperactivation, enhancing tumoral proliferation, invasiveness, and resistance to chemotherapy.

Subsequent co-immunoprecipitation (COIP) experiments in A2780-DDP and parental cells revealed stronger MAP7-CBY1 interactions in A2780-DDP cells, indicating a potential competitive mechanism against β-catenin's binding to CBY1. Moreover, a diminished interaction between β-catenin and CBY1, along with a weak MAP7-β-catenin interaction, was observed (Fig. 6D). Correspondingly, immunofluorescence analyses revealed a marked increase in the colocalization of MAP7 with CBY1 in A2780-DDP cells compared to their parental counterparts, with the quantitative data escalating from 60 % to 90 % (Fig. 6E). This supports the hypothesis that MAP7's nuclear presence, particularly its interaction with CBY1, contributes significantly to the modulation of the Wnt/β-catenin pathway and the development of cisplatin resistance in ovarian cancer cells.

## 4 Discussion

Drug-resistant cell models play a crucial role in advancing chemotherapy drug development and screening. They offer a dual advantage: assessing new drugs and exploring resistance mechanisms [22]. These models mimic clinical resistance scenarios, revealing the complex interactions between cancer cells and therapeutic drugs. Common methods for establishing cisplatin-resistant cell models include high-concentration pulse exposure (short duration/high dose) and gradual dose escalation (long duration/low dose), each offering distinct mechanisms of resistance and benefits [23]. While gradual dose escalation is more prevalent due to its higher stability and success rates over prolonged treatment, pulse exposure selects for resistant clones by applying selective pressure on innate resistance mutations. Moreover, pulse treatment closely mirrors clinical protocols, facilitating the development of resistant cell lines [24,25]. In our research, we adopted a hybrid approach integrating both pulse dosing and gradual escalation techniques to cultivate drug-resistant cell models. This hybrid strategy closely emulates clinical treatment regimens, facilitating a more realistic simulation of drug resistance development. Additionally, it enhances the likelihood of successfully establishing resistant cell lines by gradually exposing cells to higher drug concentrations.

In our study, we explored the molecular dynamics underpinning cisplatin resistance in ovarian cancer, focusing on the Wnt/β-catenin signaling pathway's role. Without active Wnt/β-catenin signaling, the APC protein collaborates with Axin, GSK-3β, and CK1α to form a "destruction complex" [26]. This complex promotes the phosphorylation of β-catenin, marking it for ubiquitination and subsequent proteasome-dependent degradation. β-catenin, a multifunctional protein, plays a key role in cancer resistance, epithelial-mesenchymal transition (EMT), and other biological processes [27]. As a core member of the Wnt signaling pathway and a component of cell adhesion molecules, β-catenin plays multiple roles in regulating cell behavior and fate [28]. The cisplatin-resistant ovarian cancer cell line A2780-DDP, developed for our investigation, demonstrated a decrease in β-catenin within the cytoplasm but an increase in its phosphorylated form in the nucleus, alongside a reduction in APC. This alteration signifies β-catenin's nuclear migration and accumulation, triggering the Wnt/β-catenin pathway activation. A comparative analysis with parental cells revealed that A2780-DDP cells possess superior migratory and invasive capabilities, and an upsurge in C-myc expression. This Wnt/β-catenin/C-myc axis activation correlates with cancer stem cells' (CSCs) function enhancement and therapeutic resistance across various cancer types [29,30]. Thus, we deduce that the nuclear relocation and accumulation of β-catenin, instigated by Wnt/β-catenin activity, are fundamental in the emergence of cisplatin resistance in ovarian cancer cells, spotlighting a potential therapeutic target to counteract resistance.

CBY1 (Chibby family member 1) acts as an antagonist of β-catenin in mammalian cells [31]. Its encoded protein directly interacts with the C-terminal region of β-catenin, inhibiting its oncogenic transcriptional activation by competing with transcription factors [32,33]. This protein plays a crucial role in the Wnt signaling pathway, essential for cell proliferation, differentiation, and cancer development. CBY1 also facilitates the nuclear export of β-catenin, further diminishing its ability to activate gene expression [31,34]. In our investigation, we identified CBY1 as a potential nuclear target of MAP7 for further exploration. In A2780-DDP cells, the interaction between MAP7 and CBY1 was augmented, while the association between β-catenin and CBY1 was weakened. Additionally, a modest interaction was noted between MAP7 and β-catenin. We postulate that heightened nuclear MAP7 expression in A2780-DDP cells competitively inhibits CBY1's suppressive effect on β-catenin, resulting in β-catenin nuclear accumulation, heightened migration, invasion capabilities, and cisplatin resistance. Our study unveils, for the first time, the pairwise interactions among MAP7, β-catenin, and CBY1, shedding light on their dynamic equilibrium and signaling within cells and offering avenues for novel cancer treatment strategies targeting these interactions. Targeting the MAP7/β-catenin/CBY1 interaction may regulate the Wnt/β-catenin signaling pathway and modulate cisplatin resistance. Moreover, this investigation may yield significant insights into the pathogenesis of other diseases, particularly those linked to disruptions in cytoskeletal dynamics and nuclear signal transduction.

Research on MAP7 (Microtubule-associated protein 7) is shedding light on its pivotal role in cancer progression and drug resistance. Studies by R Zhang et al. [35]as well as N Tang [36]have demonstrated that MAP7 promotes proliferation and migration of cervical cancer cells, and its knockdown suppresses tumor growth in mouse xenograft models. In our investigation, we found that elevated MAP7 expression in ovarian cancer (OC) correlates with poor prognosis. Moreover, MAP7 enhances OC cell proliferation and migration in vitro, consistent with observations in other cancer types. Regarding drug resistance mechanisms, Qi-Nian Wu et al. [37]revealed that HIPK3 phosphorylates MAP7, regulating its stability and contributing to platinum resistance in gastric cancer through the MEF2C–HIPK3-MAP7 axis. Additionally, in breast cancer, MAP7 upregulation is associated with paclitaxel resistance, while its downregulation inhibits cell viability, motility, and invasion, and enhances apoptosis in paclitaxel-resistant cells [18]. Our study employed a pulse and gradual dose escalation method to establish acquired cisplatin-resistant cell models, revealing a progressive increase in MAP7 protein levels in A2780-DDP cells, primarily localized in the nucleus. Current research on MAP7's nuclear function is still in its early stages. Studies by Nunu Mchedlishvili et al. [38]. found that Ensconsin/MAP7 decorates interphase microtubules in untreated HeLa cells but loses early microtubules during prophase. If Ensconsin/MAP7 is not inactivated, interphase microtubule clusters continue into metaphase, disrupting spindle assembly. Sestina Falcone et al. [39]. showed that peripheral nuclear positioning requires microtubule/Map7/Kif5b-dependent nuclear distribution along myofibers, driven by actin and Nesprin. The exploration of MAP7's activities within the nucleus and its contribution to cellular processes represents a burgeoning area of scientific inquiry, crucial for unraveling the complex regulatory mechanisms governing nuclear structure and function. In our current study, we observed an upregulation and predominant nuclear localization of MAP7 in cisplatin-resistant A2780-DDP ovarian cancer cells, implicating its significant role in mediating drug resistance. Furthermore, we pinpointed CBY1 as a promising nuclear target of MAP7, warranting deeper investigation. This finding not only contributes to our understanding of MAP7's functional repertoire but also highlights its potential as a pivotal factor in cancer drug resistance, opening avenues for targeted therapeutic strategies against ovarian cancer and possibly other malignancies exhibiting chemoresistance.

Chemotherapy resistance and metastasis pose formidable challenges to effective cancer treatment [40]. Epithelial-mesenchymal transition (EMT) plays a pivotal role in cancer cell migration, metastasis, and resistance to cisplatin [41]. During EMT, epithelial cells lose their typical adhesion properties, gain enhanced migratory and invasive capabilities, and develop resistance to chemotherapy [42]. Our findings suggest promising therapeutic avenues for MAP7. Combination therapy involving cisplatin can target the EMT process, overcoming cisplatin resistance and hindering tumor migration and metastasis. MAP7, as a biomarker linked to cisplatin resistance, holds promise for predicting patient responses to cisplatin treatment, thereby enabling personalized therapy. Developing small molecule inhibitors targeting MAP7 directly to curb its function in ovarian cancer cells may offer novel drug targets for tackling cisplatin-resistant ovarian cancer. However, the lack of confirmatory experiments and animal validation represents a limitation of this study and warrants further research and efforts.

## 5 Conclusion

In summary, our research sheds light on CBY1 as a crucial nuclear target for MAP7 within cisplatin-resistant A2780-DDP ovarian cancer cells. Through inhibiting the CBY1-β-catenin interaction, we uncovered a new pathway by which MAP7 augments β-catenin's transcriptional activity and nuclear presence, significantly influencing the EMT phenotype and cisplatin resistance in A2780-DDP cells. These findings provide a deeper understanding of the complex mechanisms behind cisplatin resistance, offering a promising direction for developing targeted therapies to counteract this prevalent challenge in ovarian cancer treatment.

## Ethics and consent section

All specimens involved in this study were tumor-related tissues excised during ovarian cancer surgeries. Oral consent was obtained from all patients involved, permitting the use of these specimens for scientific research purposes.The Ethical Committee of Shanghai Fengxian District Central Hospital reviewed and approved the collection of samples and the design of the experiment in this study(2017-KY-07).

## Funding

This study was supported by grants from Fengxian District Science and Technology Commission Project (no.20221407 to QH Huang).

## Data availability statement

The RNA-seq data have been submitted to the China National Center for Bioinformation, National Genomics Data Center (https://ngdc.cncb.ac.cn/gsa-human/browse/HRA006942) (accession number HRA009931).

## CRediT authorship contribution statement

**Qingqing Chen:** Writing – original draft, Visualization, Validation, Software, Project administration, Methodology, Formal analysis, Data curation, Conceptualization. **Shaojing Li:** Validation, Software, Methodology, Investigation, Formal analysis, Data curation, Conceptualization. **Furong Fu:** Validation, Software, Resources, Investigation, Formal analysis, Data curation, Conceptualization. **Qunhuan Huang:** Writing – review & editing, Supervision, Resources, Funding acquisition, Data curation, Conceptualization. **Rong Zhang:** Writing – review & editing, Validation, Supervision, Resources, Methodology, Data curation, Conceptualization.

## Declaration of competing interest

The authors declare that they have no known competing financial interests or personal relationships that could have appeared to influence the work reported in this paper.
