# Supplementary figures and images for: MAP7 drives EMT and cisplatin resistance in ovarian cancer via wnt/β-catenin signaling
Source: Heliyon. 2024 Apr 29;10(9):e30409. doi: 10.1016/j.heliyon.2024.e30409 (PMC11078642; doi:10.1016/j.heliyon.2024.e30409)

# KEGG enrichment analysis(caov3\_s1\_vs\_caov3\_nc\_all\_1)

KEGG pathway

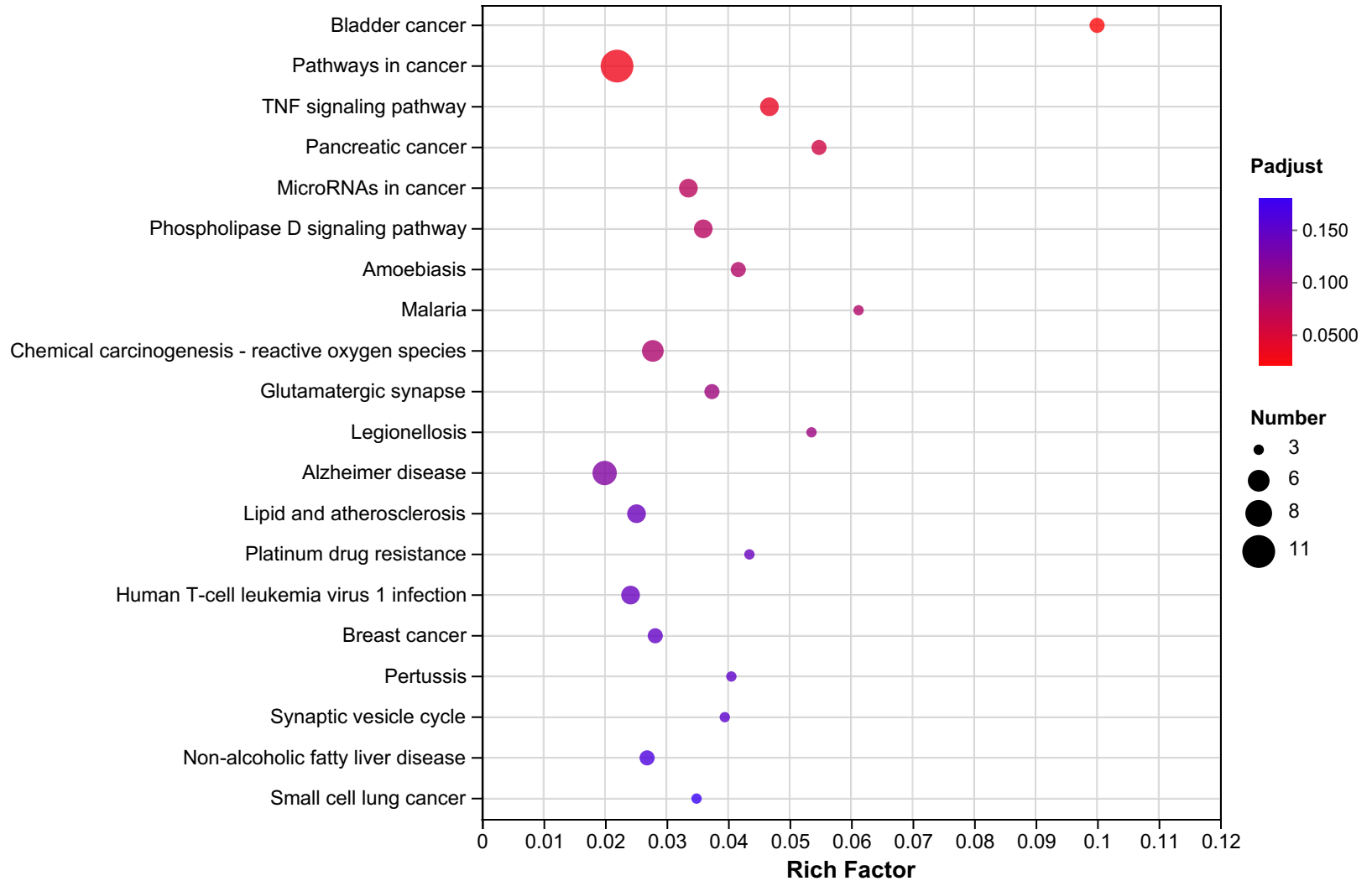

Supplement: Multimedia component 1 [file mmc1.pdf]
