# Supplementary material for: MAP7 drives EMT and cisplatin resistance in ovarian cancer via wnt/β-catenin signaling
Source: Heliyon. 2024 Apr 29;10(9):e30409. doi: 10.1016/j.heliyon.2024.e30409 (PMC11078642; doi:10.1016/j.heliyon.2024.e30409)
Supplement: Multimedia component 2 [file mmc2.pdf]

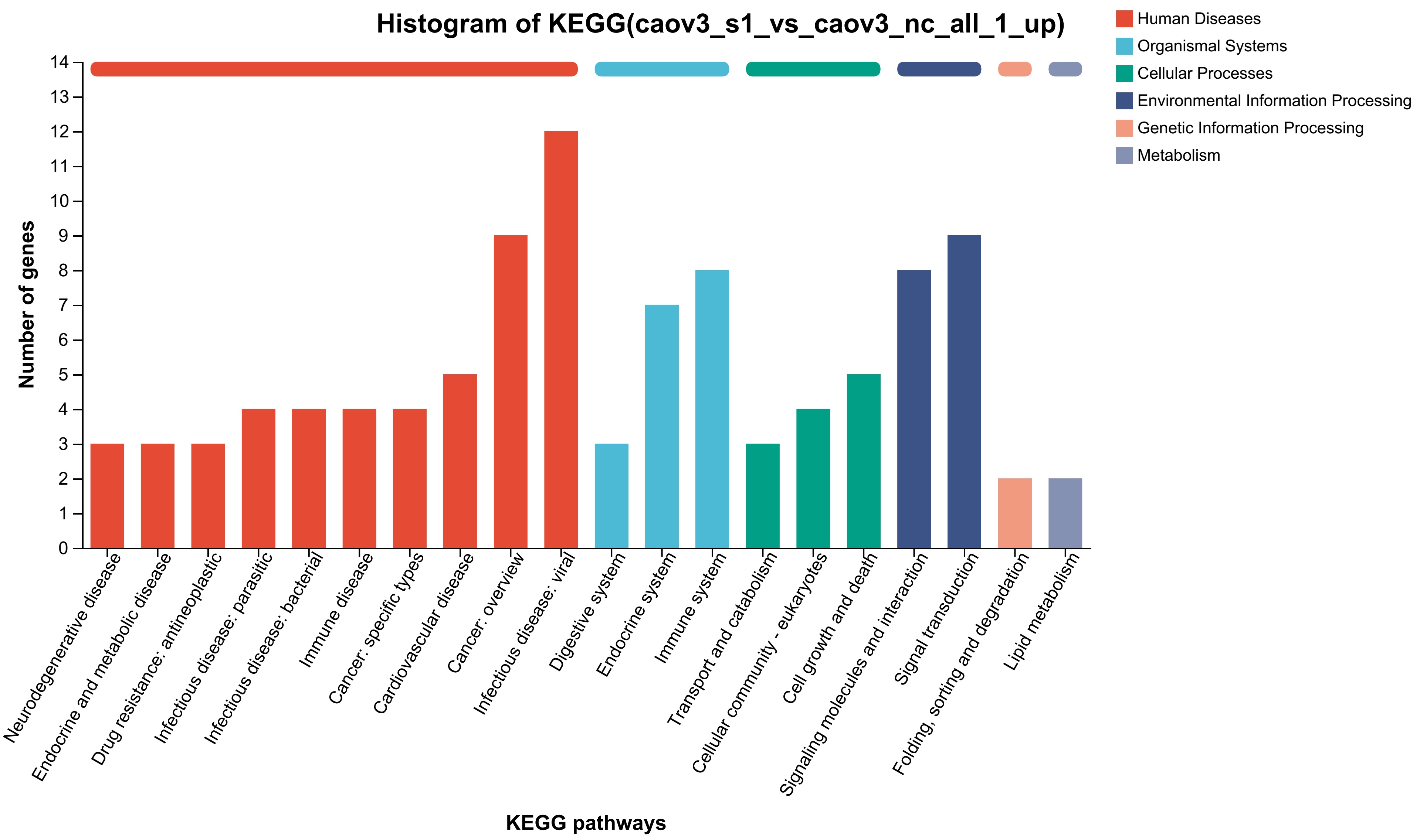

Histogram of KEGG(caov3_s1_vs_caov3_nc_all_1_up)
Human Diseases
Organismal Systems
Cellular Processes
Environmental Information Processing
Genetic Information Processing
Metabolism
Number of genes
0
1
2
3
4
5
6
7
8
9
10
11
12
13
14
Neurodegenerative disease
Endocrine and metabolic disease
Drug resistance: antineoplastic
Infectious disease: parasitic
Infectious disease: bacterial
Immune disease
Cancer: specific types
Cardiovascular disease
Cancer: overview
Infectious disease: viral
Digestive system
Endocrine system
Immune system
Transport and catabolism
Cellular community - eukaryotes
Cell growth and death
Signaling molecules and interaction
Signal transduction
Folding, sorting and degradation
Lipid metabolism
KEGG pathways
