# Supplementary material for: MAP7 drives EMT and cisplatin resistance in ovarian cancer via wnt/β-catenin signaling
Source: Heliyon. 2024 Apr 29;10(9):e30409. doi: 10.1016/j.heliyon.2024.e30409 (PMC11078642; doi:10.1016/j.heliyon.2024.e30409)
Supplement: Multimedia component 3 [file mmc3.pdf]

Figure 2B

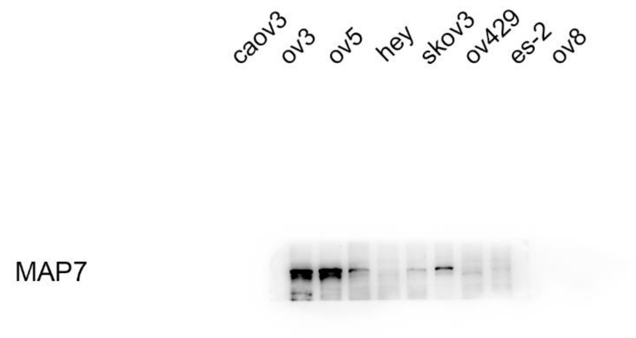
caov3
ov3
ov5
hey
skov3
ov429
es-2
ov8
MAP7

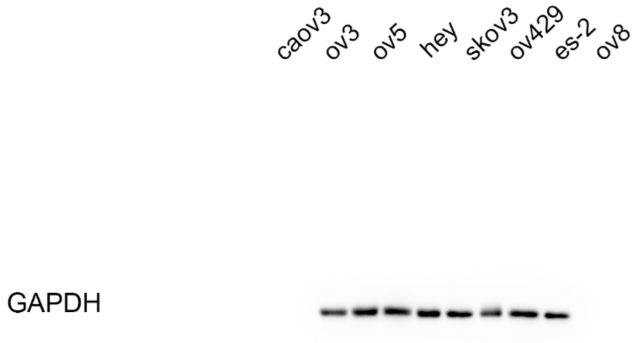
caov3
ov3
ov5
hey
skov3
ov429
es-2
ov8
GAPDH

Figure 2C

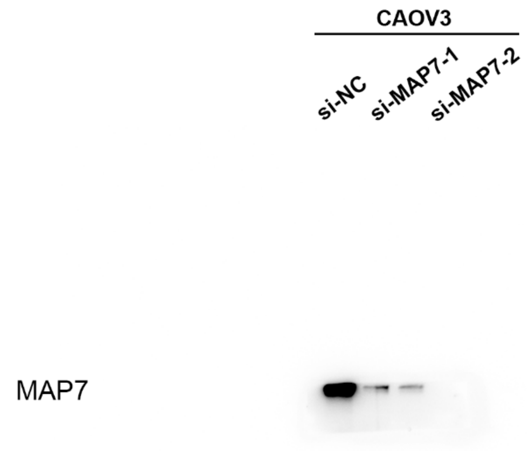
CAOV3
si-NC
si-MAP7-1
si-MAP7-2
MAP7

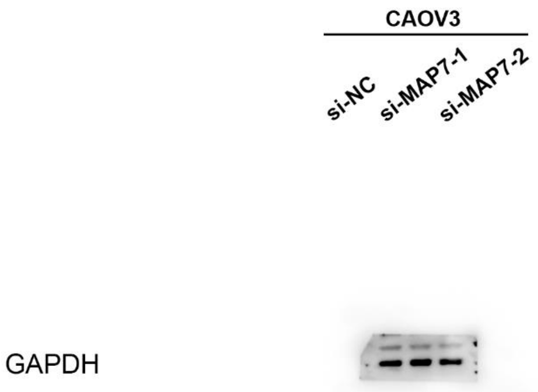
CAOV3
si-NC
si-MAP7-1
si-MAP7-2
GAPDH

Figure 2E

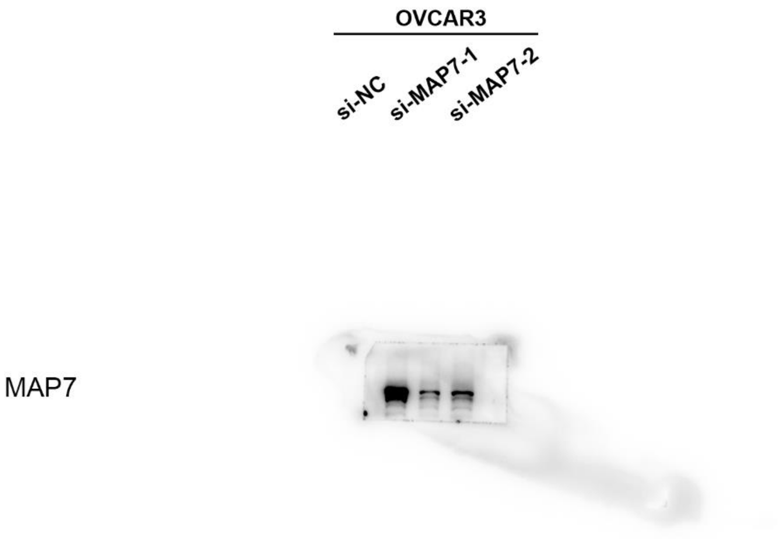

OVCAR3
si-NC
si-MAP7-1
si-MAP7-2
MAP7

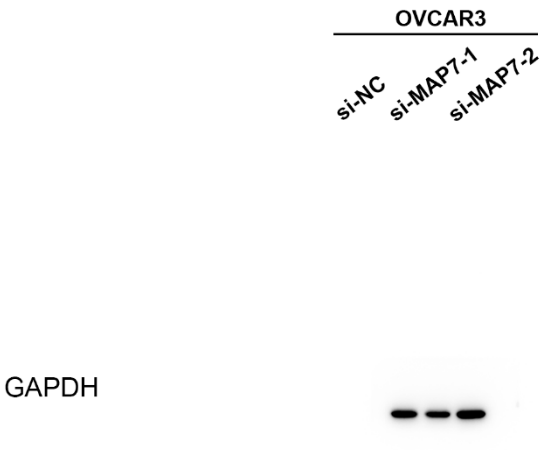

OVCAR3
si-NC
si-MAP7-1
si-MAP7-2
GAPDH

Figure 3A

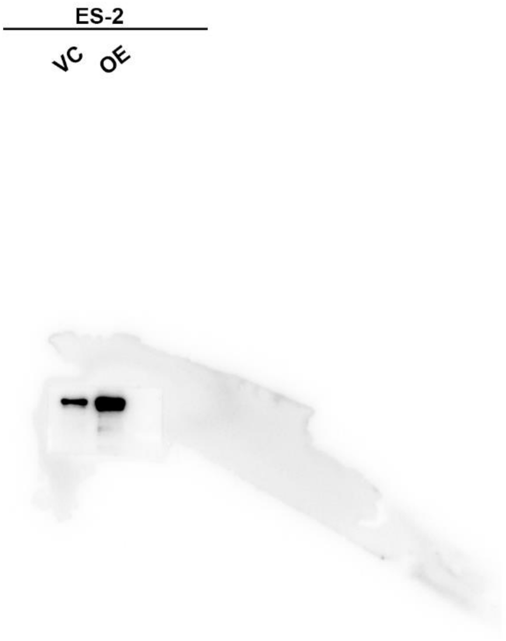

MAP7

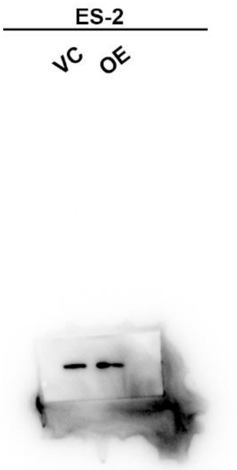

GAPDH

Figure 3B

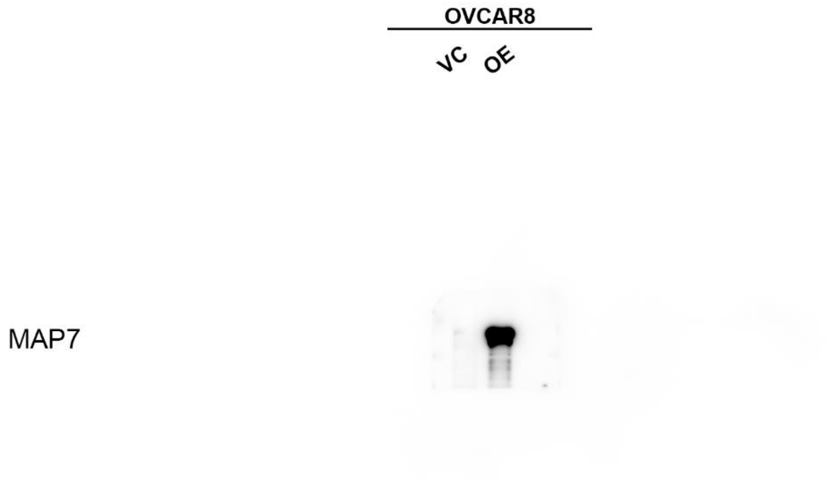

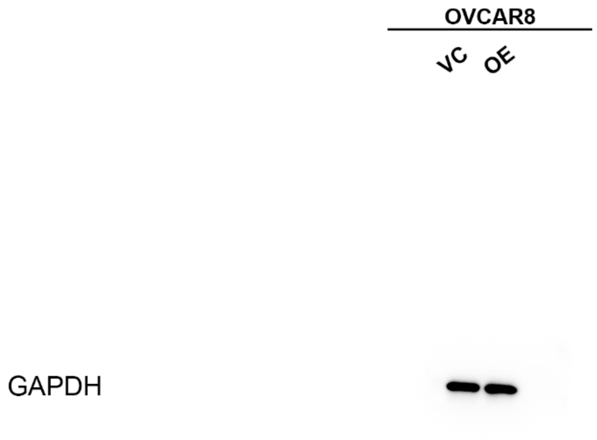

Figure 4C-L

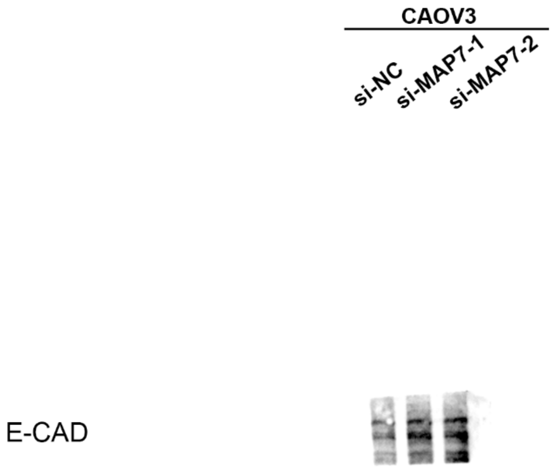
CAOV3
si-NC
si-MAP7-1
si-MAP7-2
E-CAD

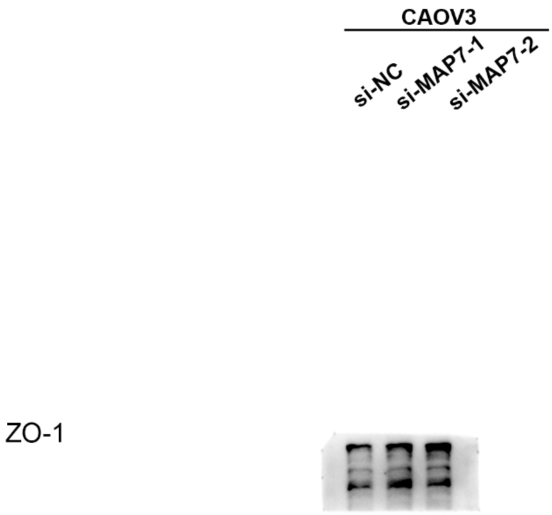
CAOV3
si-NC
si-MAP7-1
si-MAP7-2
ZO-1

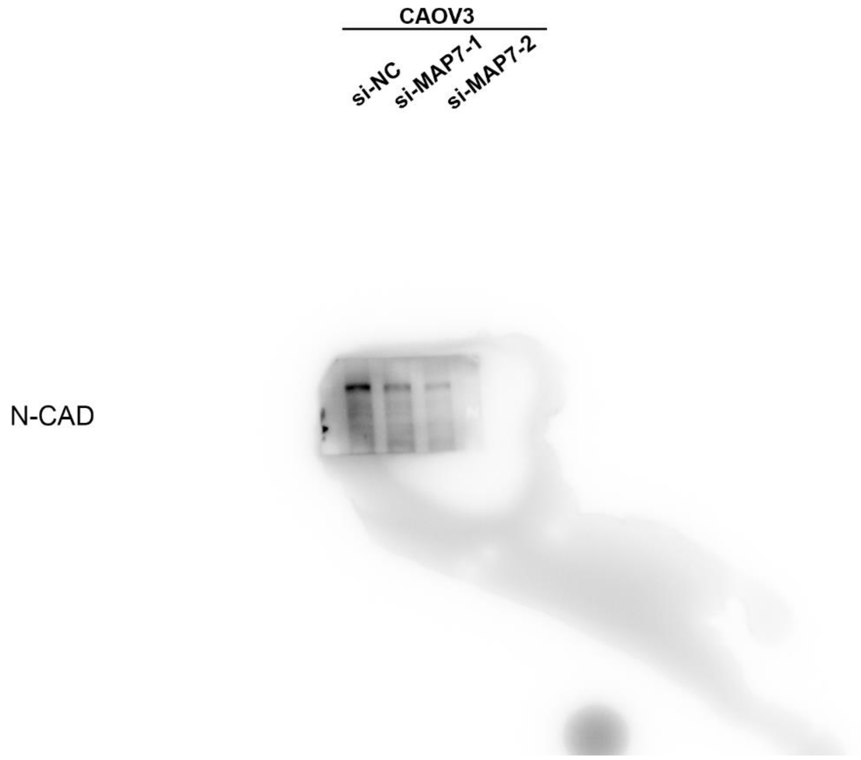
CAOV3
si-NC
si-MAP7-1
si-MAP7-2
N-CAD

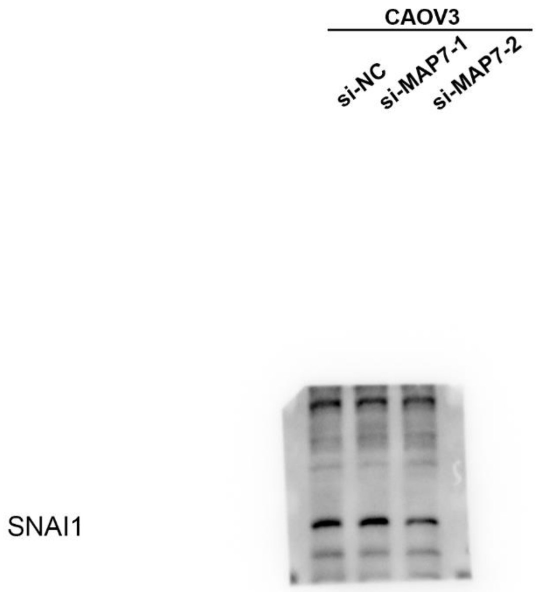
CAOV3
si-NC
si-MAP7-1
si-MAP7-2
SNAI1

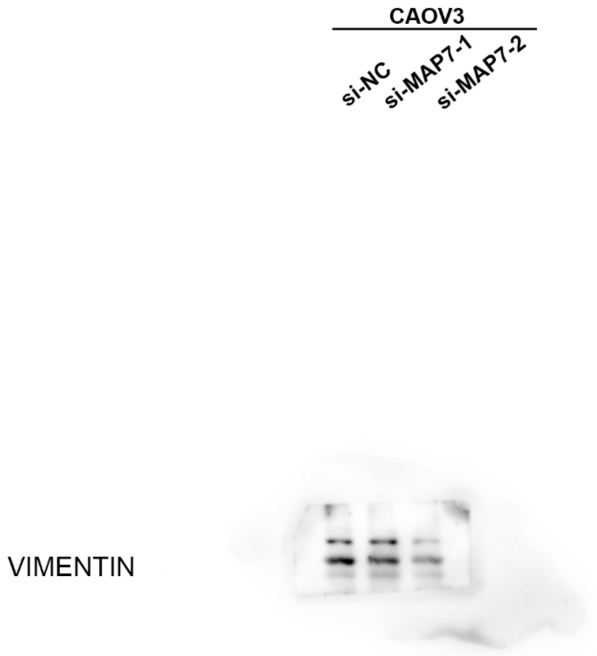
CAOV3
si-NC
si-MAP7-1
si-MAP7-2
VIMENTIN

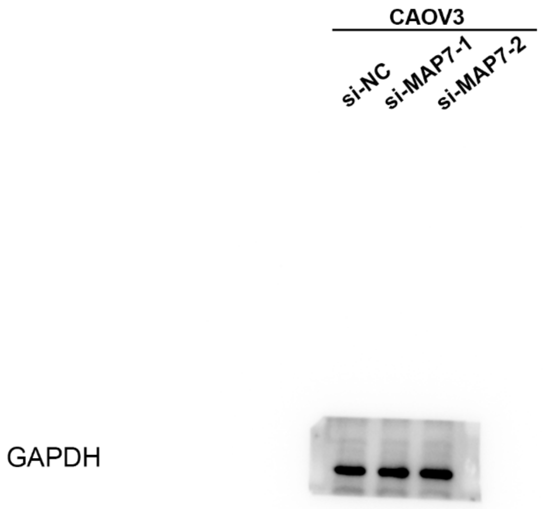
CAOV3
si-NC
si-MAP7-1
si-MAP7-2
GAPDH

Figure 4C-R

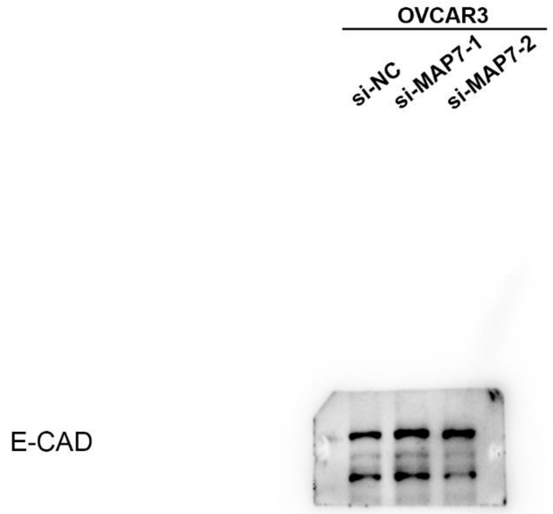
OVCAR3
si-NC
si-MAP7-1
si-MAP7-2
E-CAD

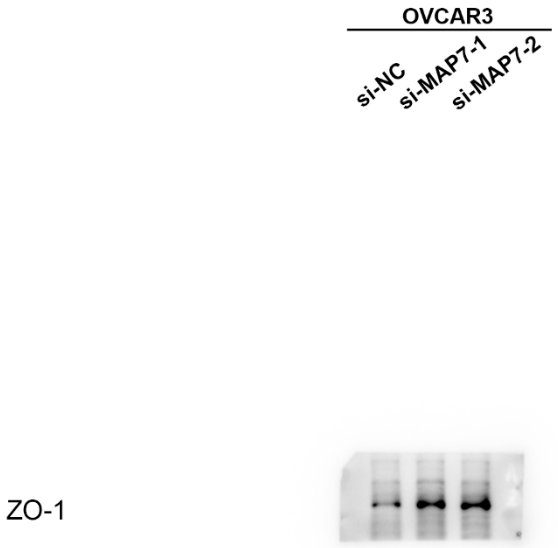
OVCAR3
si-NC
si-MAP7-1
si-MAP7-2
ZO-1

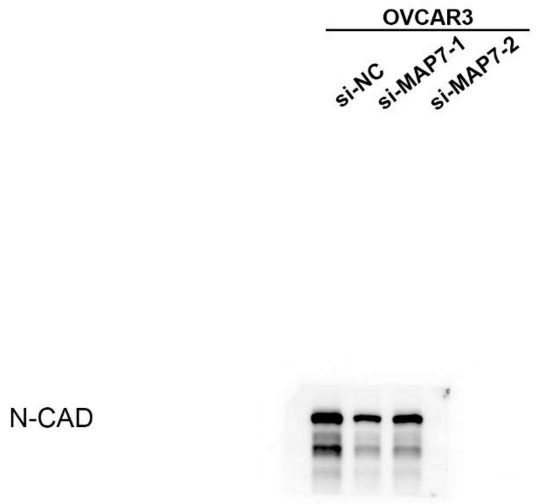
OVCAR3
si-NC
si-MAP7-1
si-MAP7-2
N-CAD

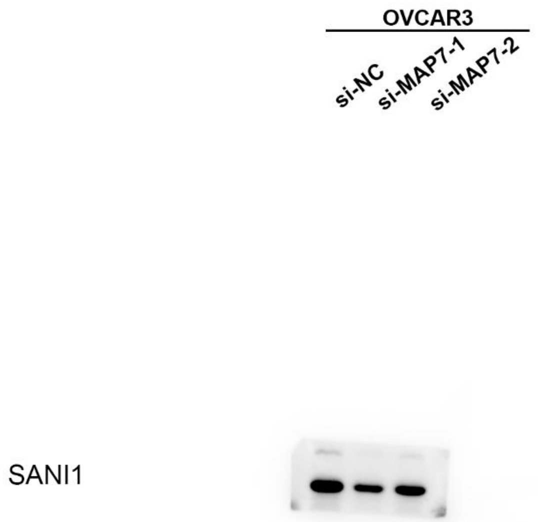
OVCAR3
si-NC
si-MAP7-1
si-MAP7-2
SANI1

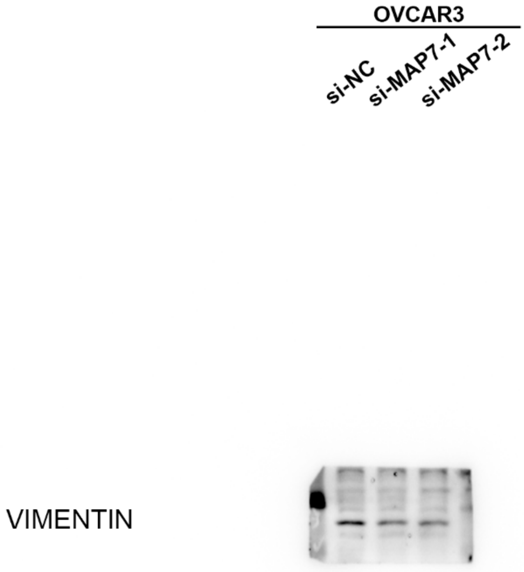
OVCAR3
si-NC
si-MAP7-1
si-MAP7-2
VIMENTIN

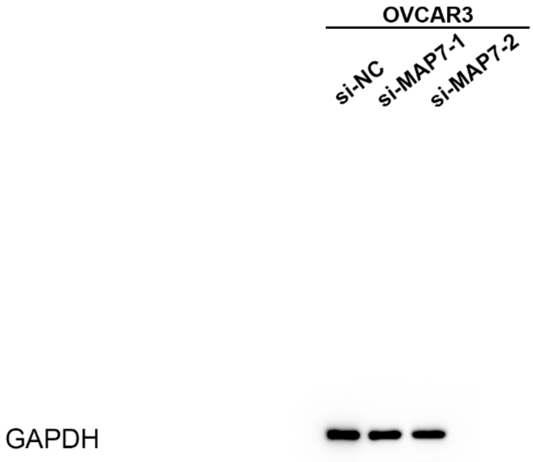
OVCAR3
si-NC
si-MAP7-1
si-MAP7-2
GAPDH

Figure 4D-L

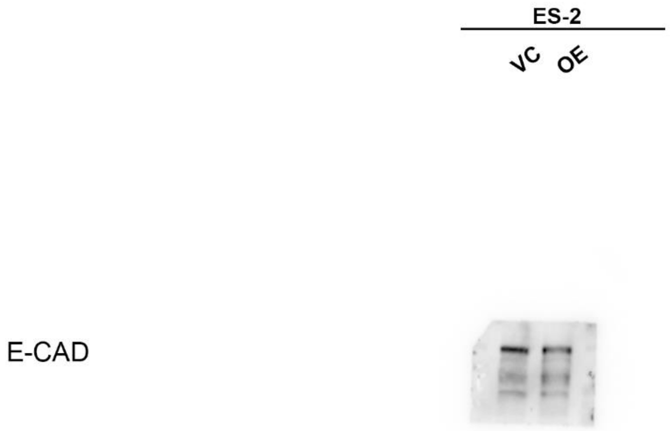

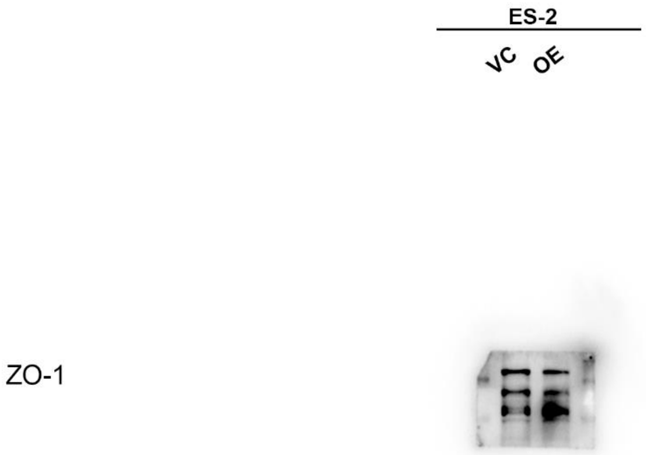

ES-2

VC OE

N-CAD

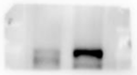

ES-2

VC OE

SANI1

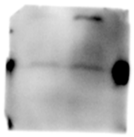

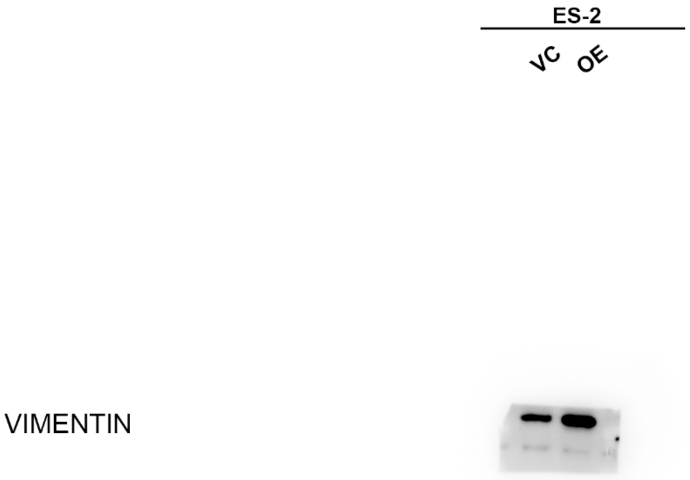
ES-2
VC
OE
VIMENTIN

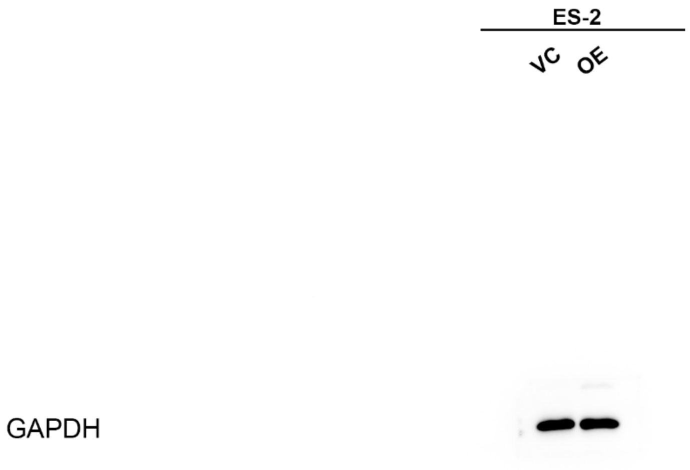
ES-2
VC
OE
GAPDH

Figure 4D-R

OVCAR8

VC OE

E-CAD

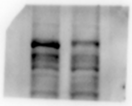

OVCAR8

VC OE

ZO-1

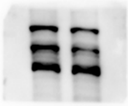

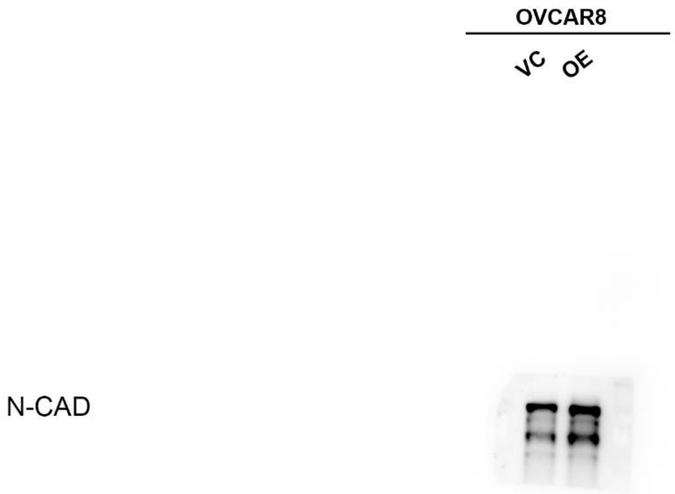
OVCAR8
VC
OE
N-CAD

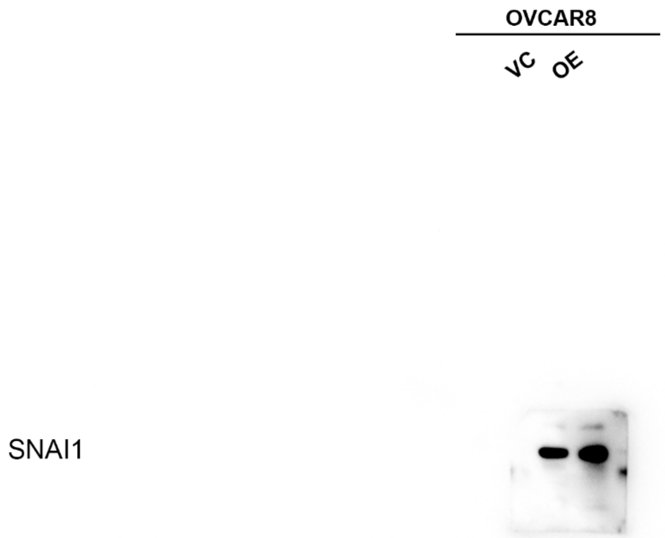
OVCAR8
VC
OE
SNAI1

OVCAR8

VC OE

VIMENTIN

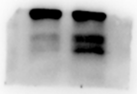

OVCAR8

VC OE

GAPDH

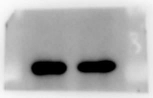

Figure 4E-L

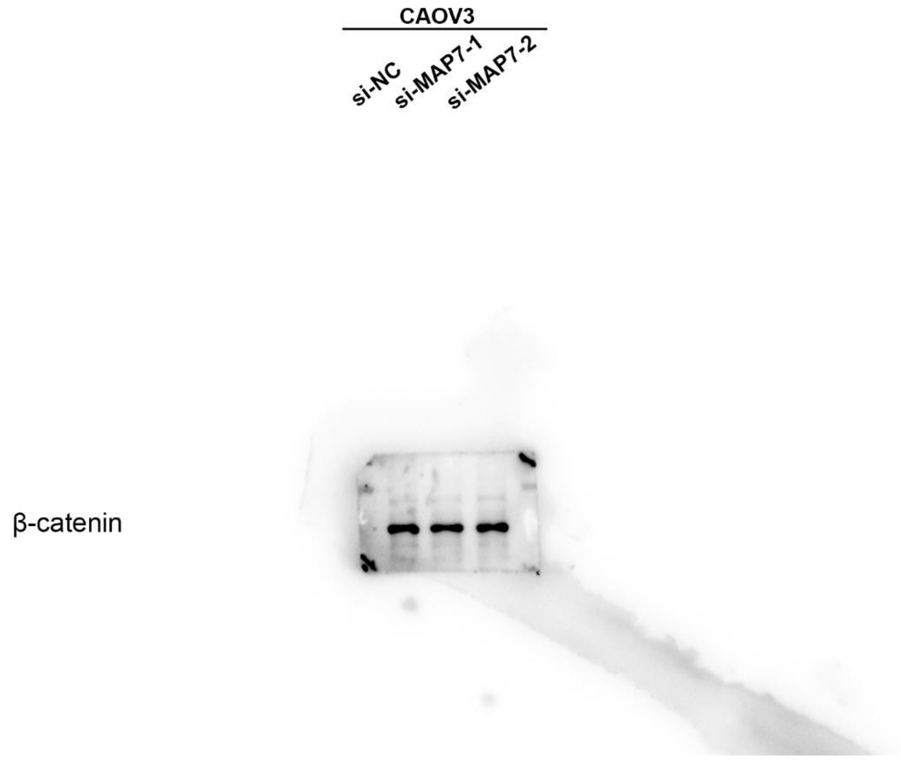
CAOV3
si-NC
si-MAP7-1
si-MAP7-2
β-catenin

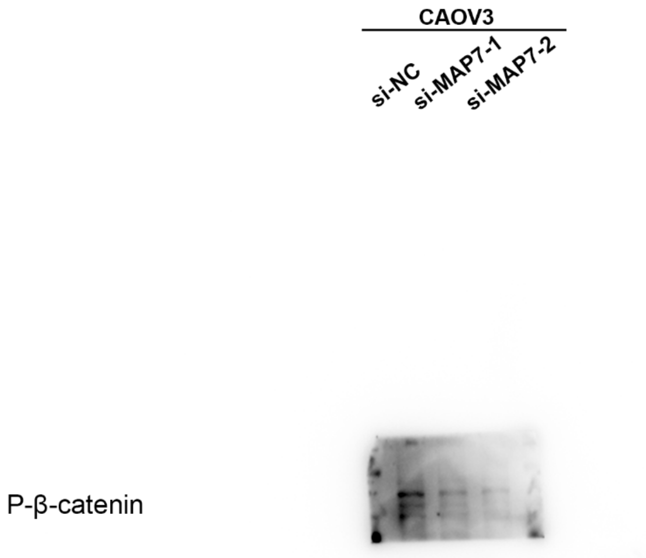
CAOV3
si-NC
si-MAP7-1
si-MAP7-2
P-β-catenin

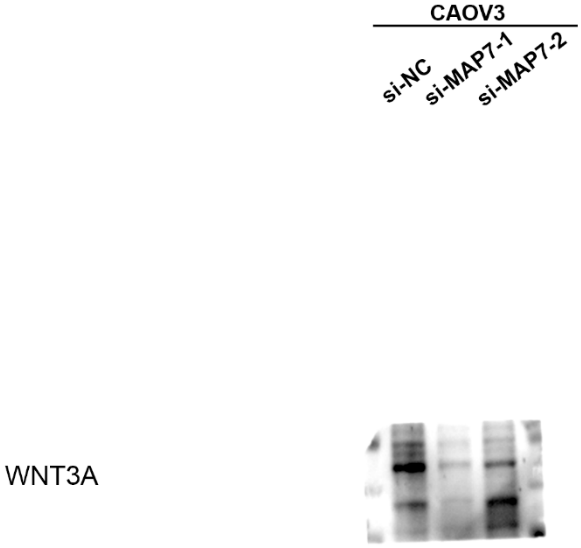
CAOV3
si-NC
si-MAP7-1
si-MAP7-2
WNT3A

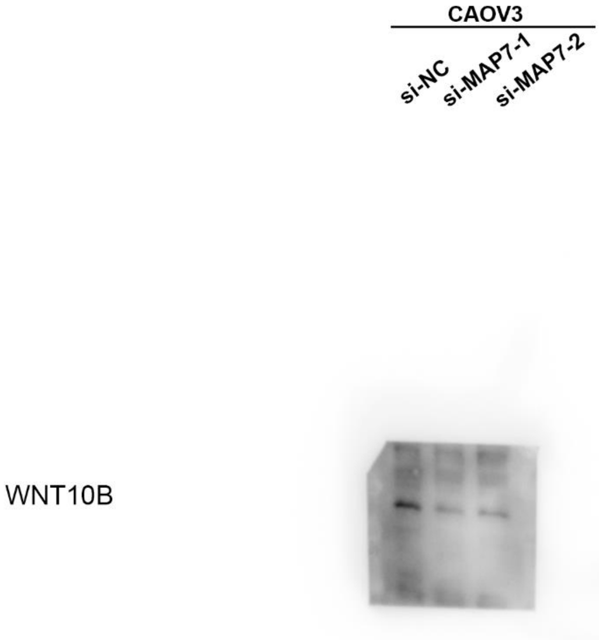
CAOV3
si-NC
si-MAP7-1
si-MAP7-2
WNT10B

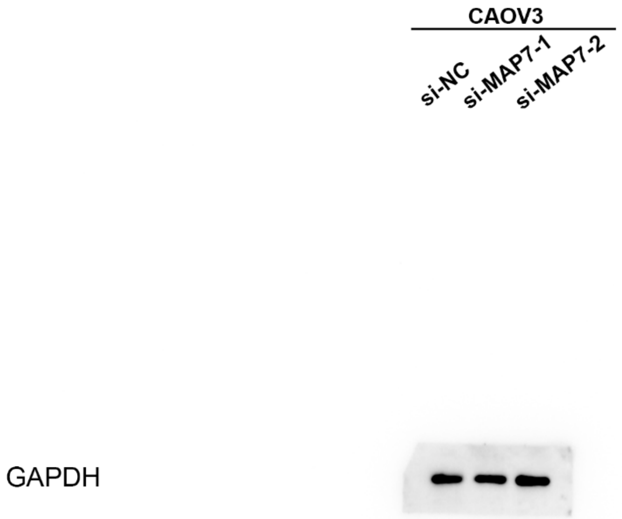
CAOV3
si-NC
si-MAP7-1
si-MAP7-2
GAPDH

Figure 4E-R

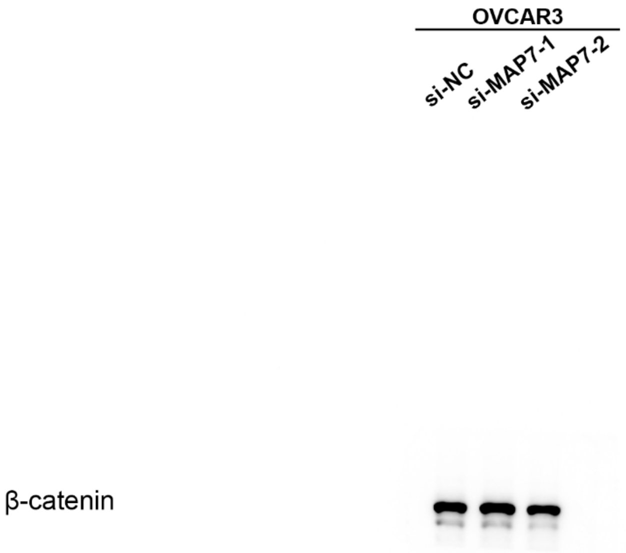
OVCAR3
si-NC
si-MAP7-1
si-MAP7-2
β-catenin

OVCAR3

si-NC si-MAP7-1 si-MAP7-2

P-β-catenin

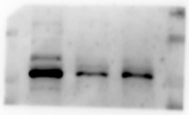

OVCAR3

si-NC si-MAP7-1 si-MAP7-2

WNT3A

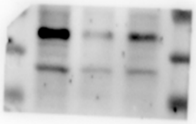

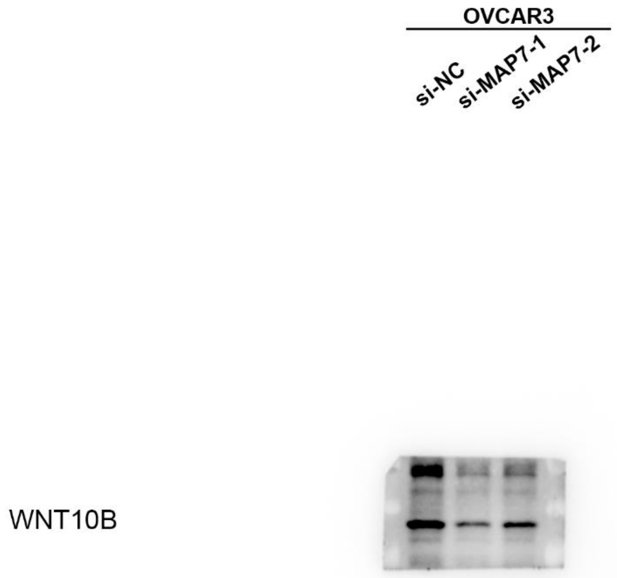
OVCAR3
si-NC
si-MAP7-1
si-MAP7-2
WNT10B

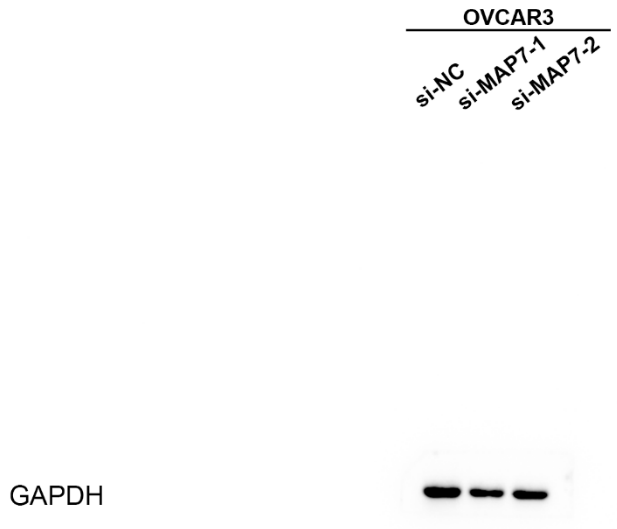
OVCAR3
si-NC
si-MAP7-1
si-MAP7-2
GAPDH

Figure 4F-L

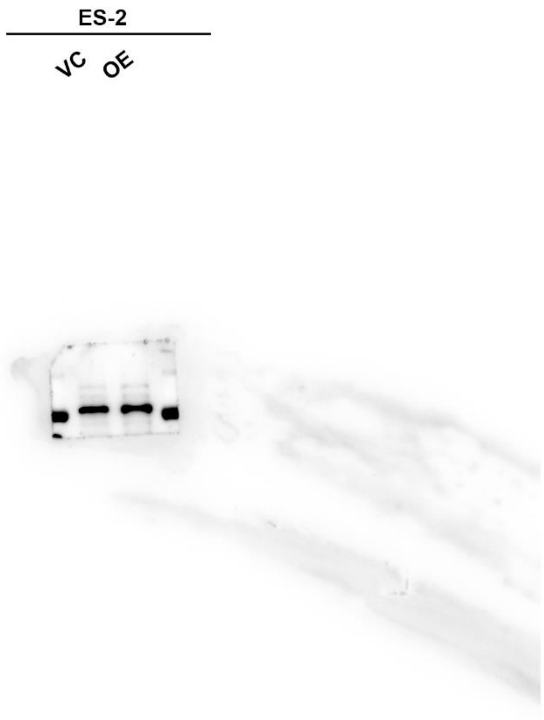

β-catenin

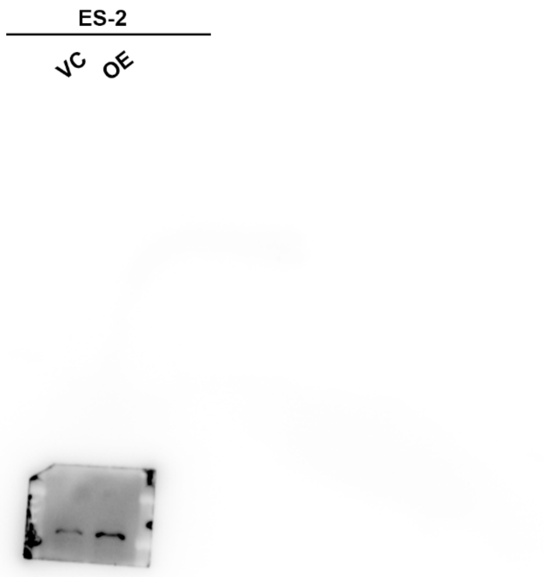

P-β-catenin

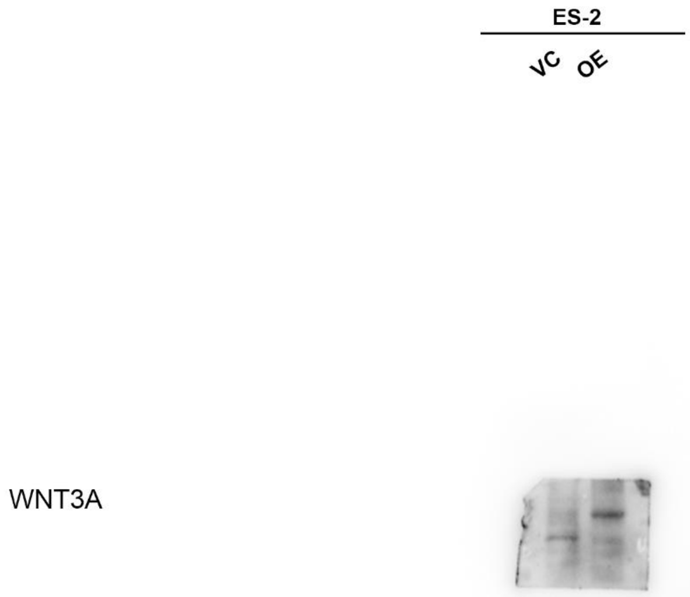
ES-2
VC
OE
WNT3A

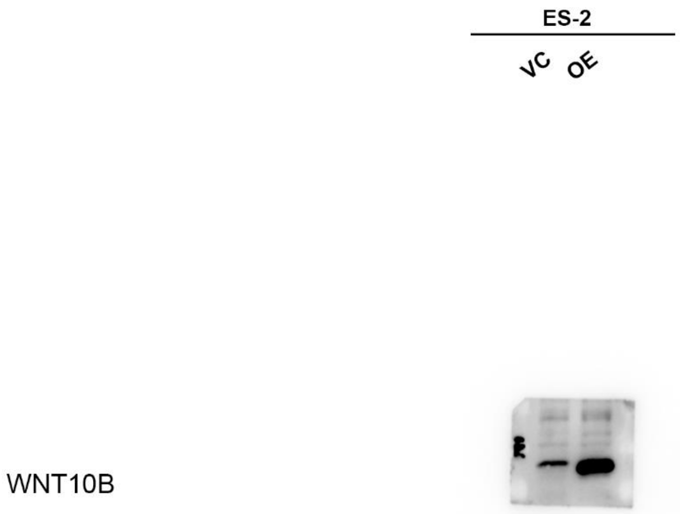
ES-2
VC
OE
WNT10B

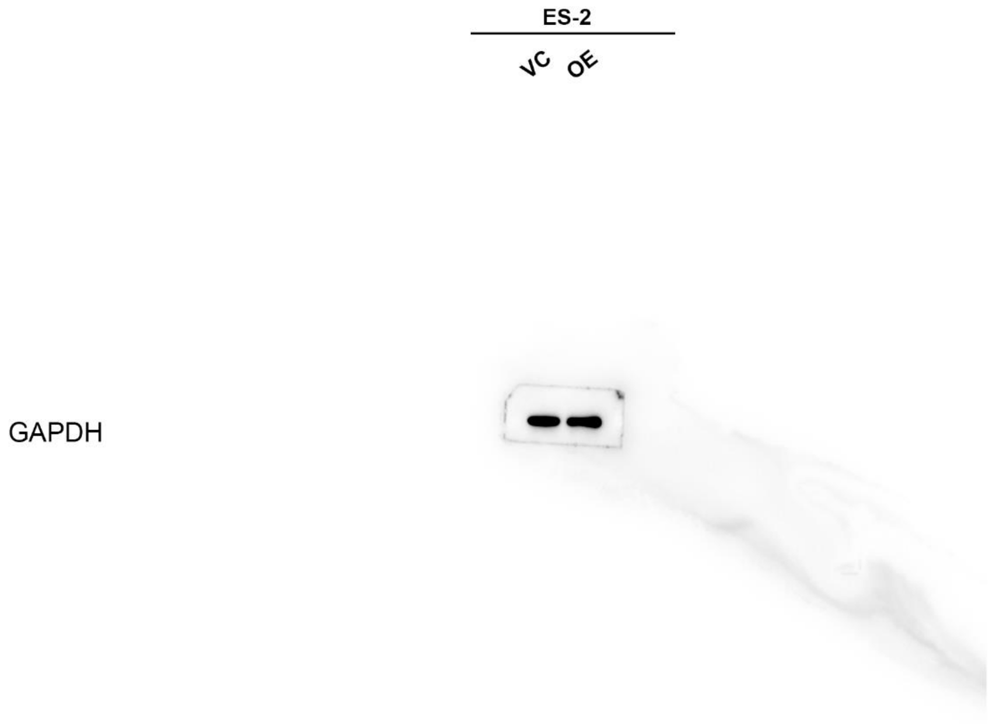

Figure 4F-R

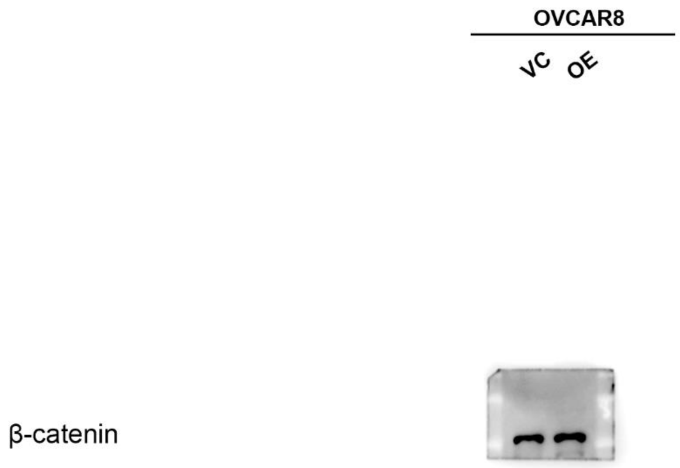

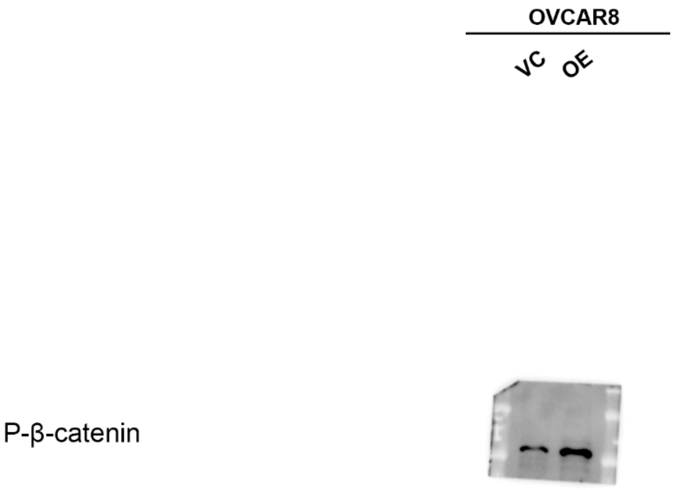
OVCAR8
VC
OE
P-β-catenin

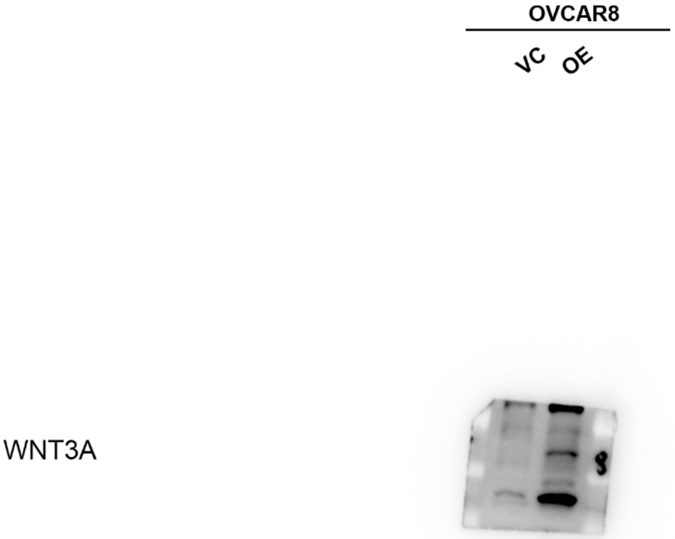
OVCAR8
VC
OE
WNT3A

OVCAR8

VC OE

WNT10B

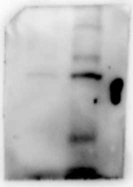

OVCAR8

VC OE

GAPDH

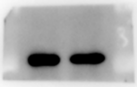

Figure 5C-L

MAP7

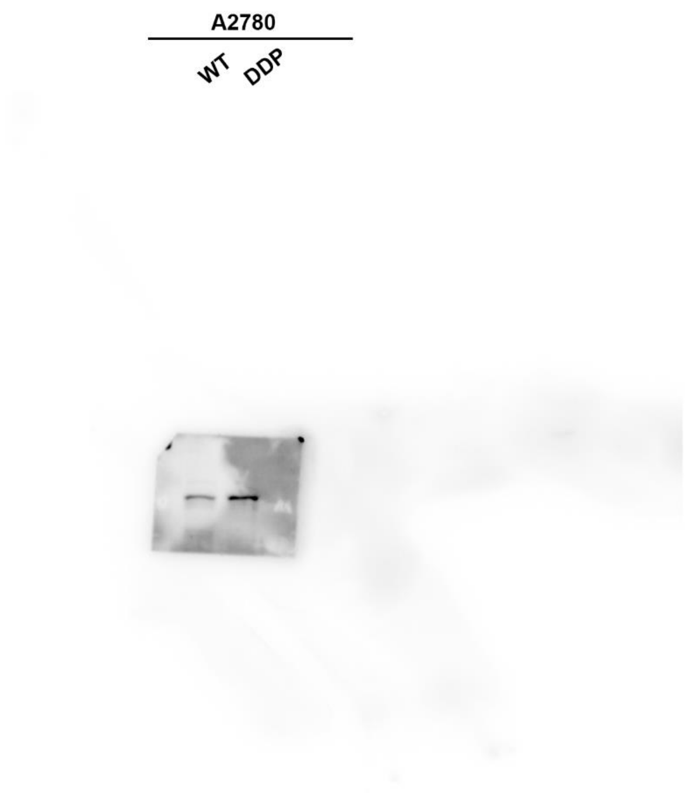

GAPDH

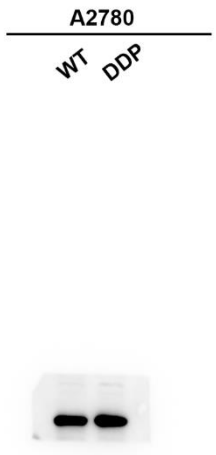

Figure 5C-M

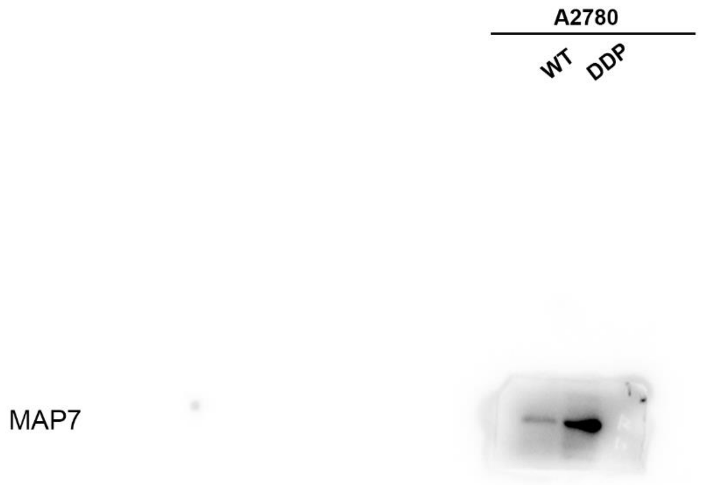

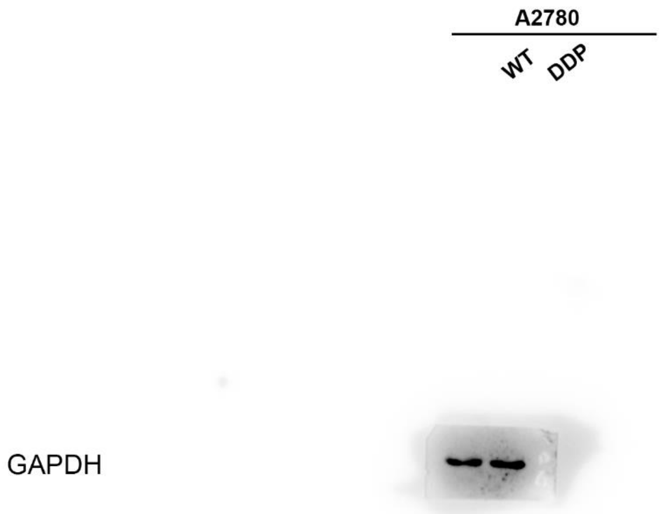

Figure 5C-R

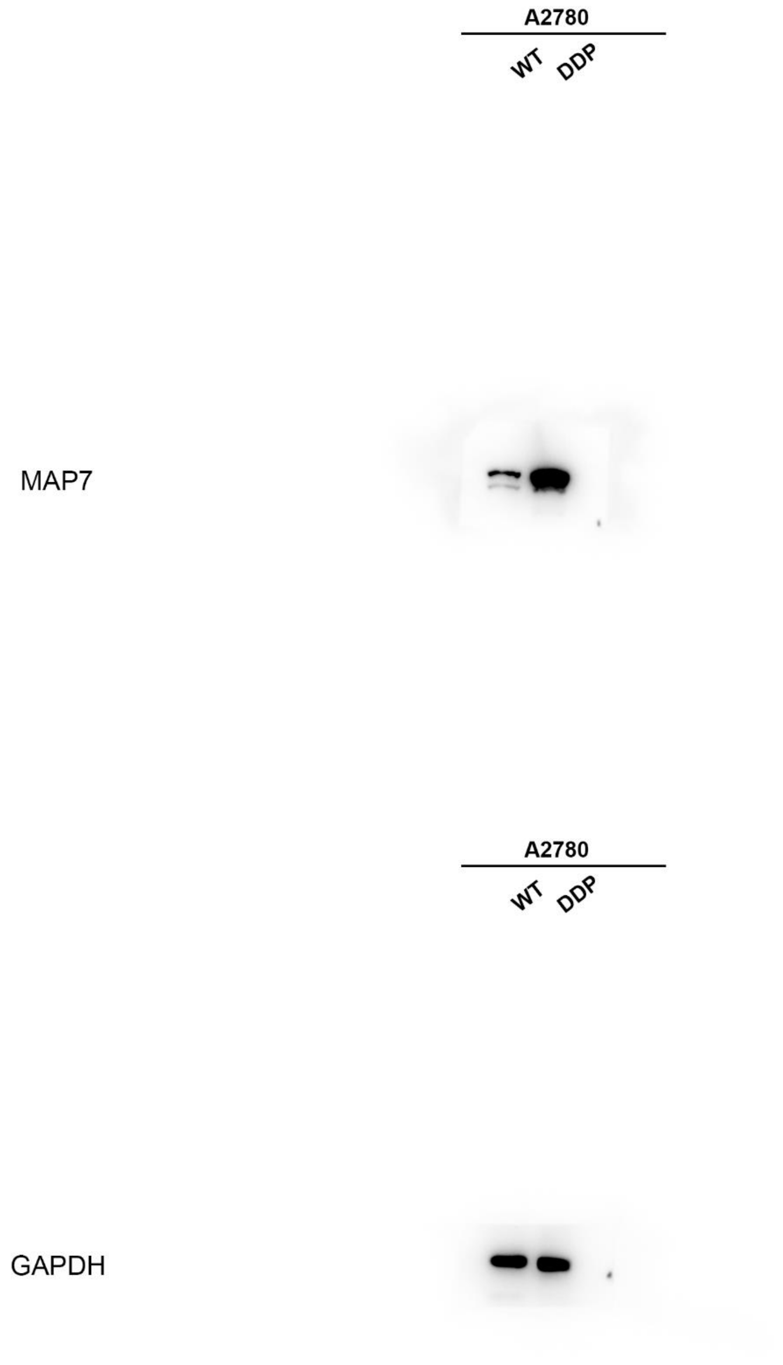

Figure 5E

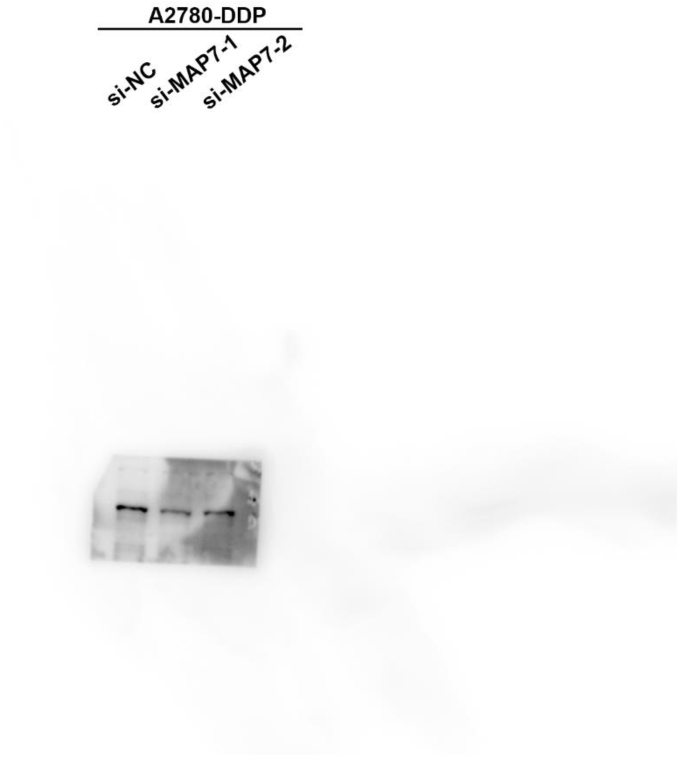

MAP7

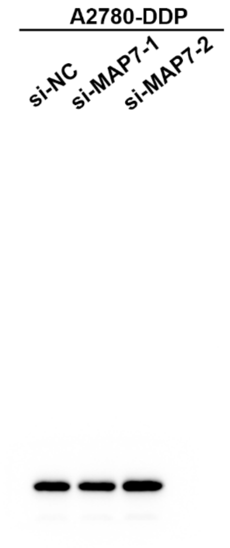

GAPDH

Figure 5H

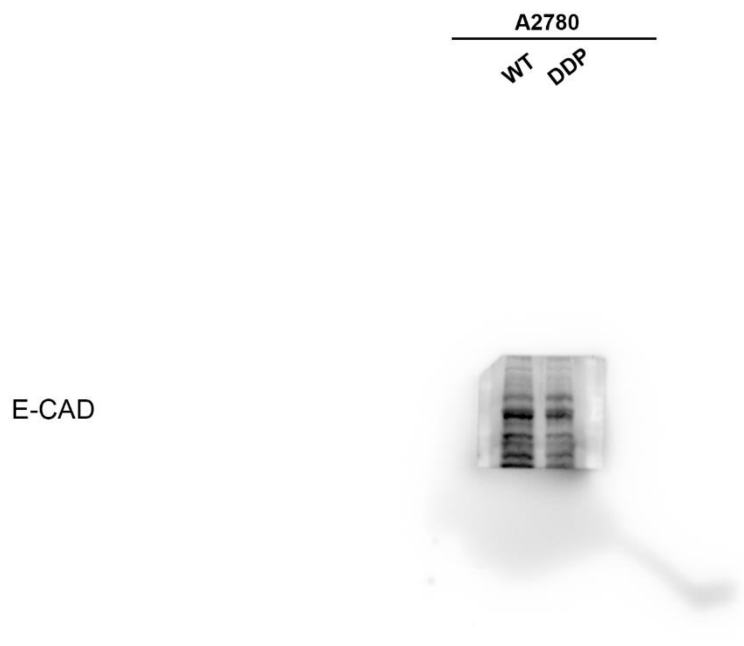

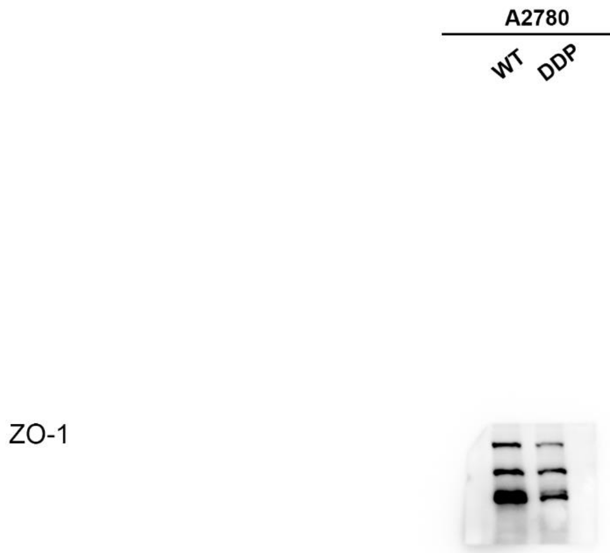

A2780

WT DDP

N-CAD

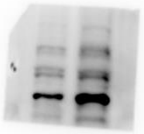

A2780

WT DDP

SNAI1

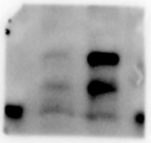

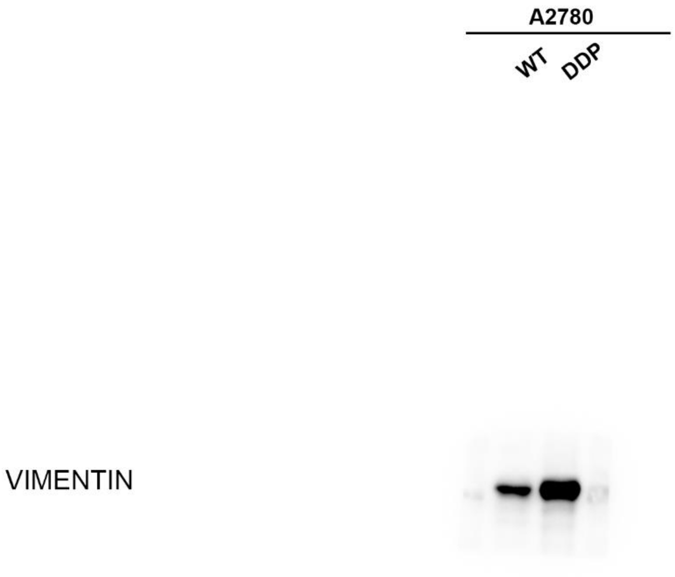
A2780
WT
DDP
VIMENTIN

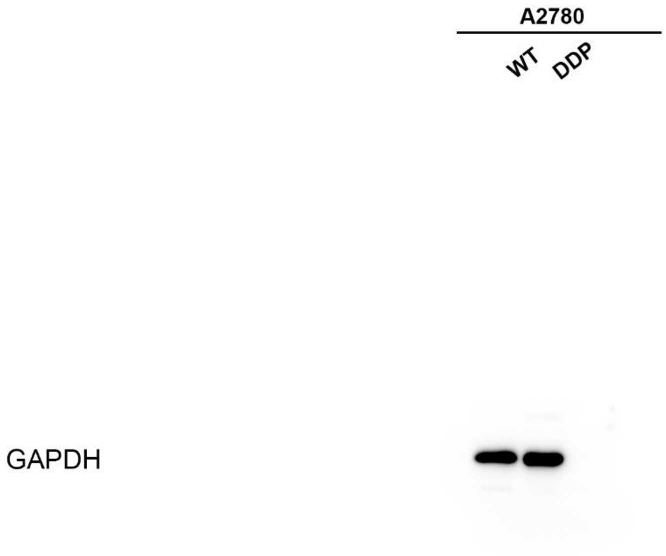
A2780
WT
DDP
GAPDH

Figure 5I

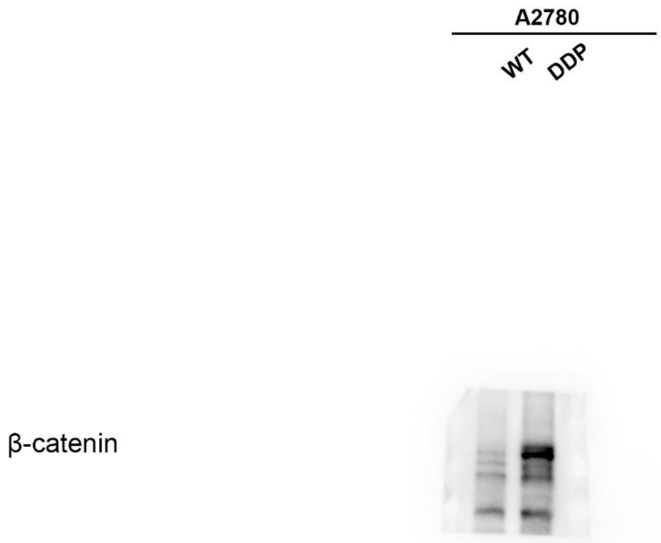

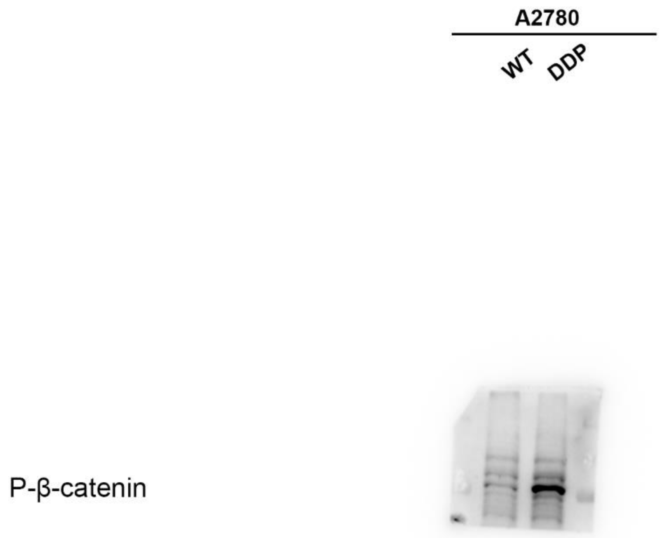

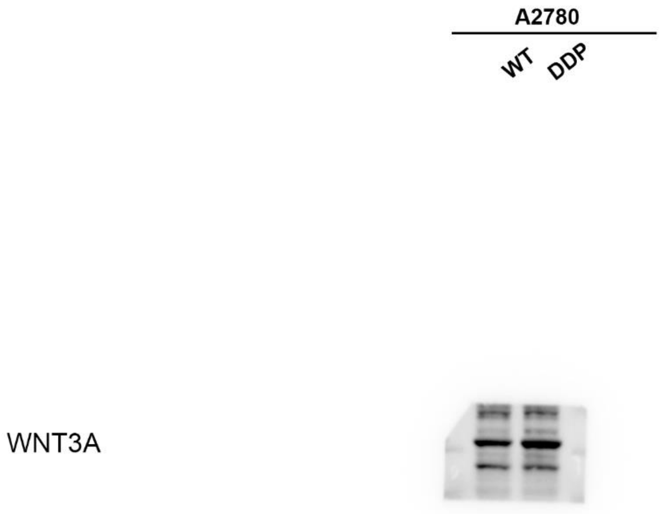
A2780
WT
DDP
WNT3A

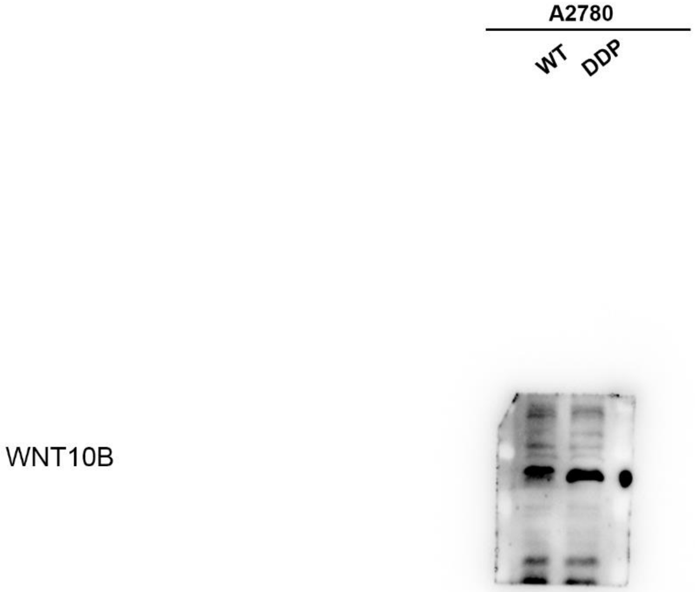
A2780
WT
DDP
WNT10B

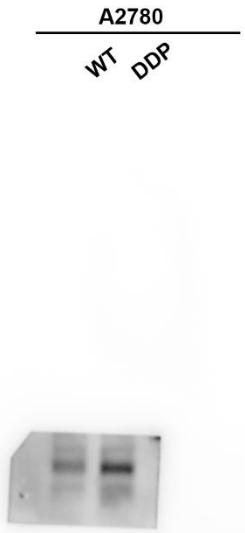

C-MYC

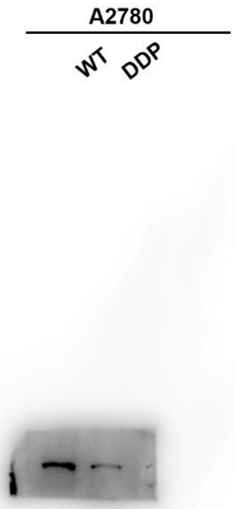

APC

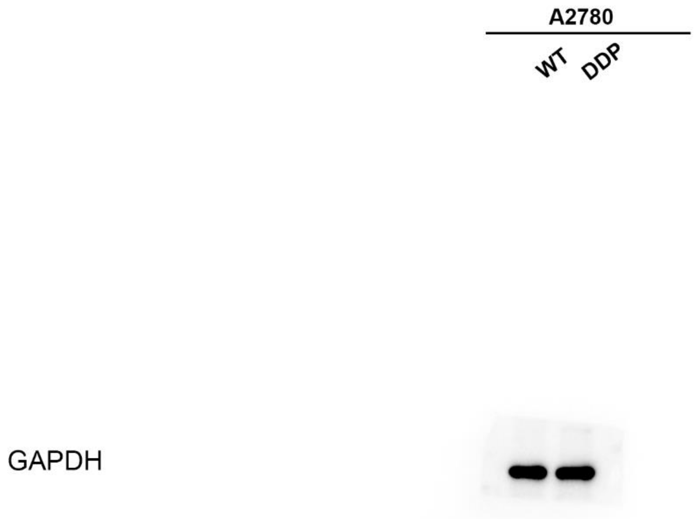
A2780
WT
DDP
GAPDH

Figure 6B-L

A2780-CYTO

WT DDP

β-catenin

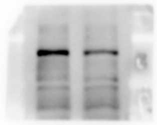

A2780-CYTO

WT DDP

P-β-catenin

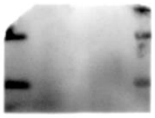

A2780-CYTO

WT DDP

MAP7

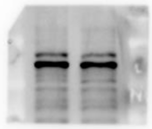

A2780-CYTO

WT DDP

GAPDH

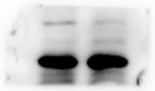

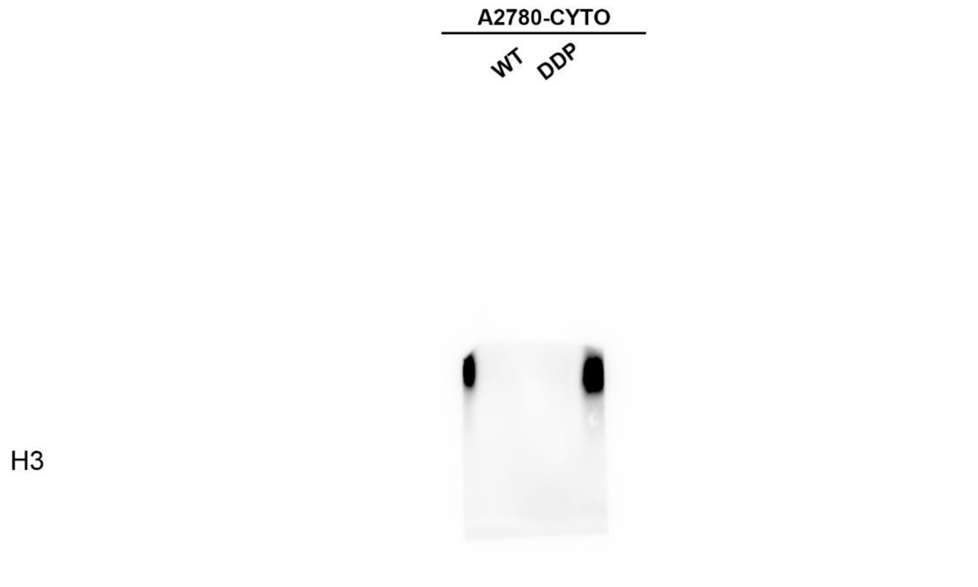

Figure 6B-R

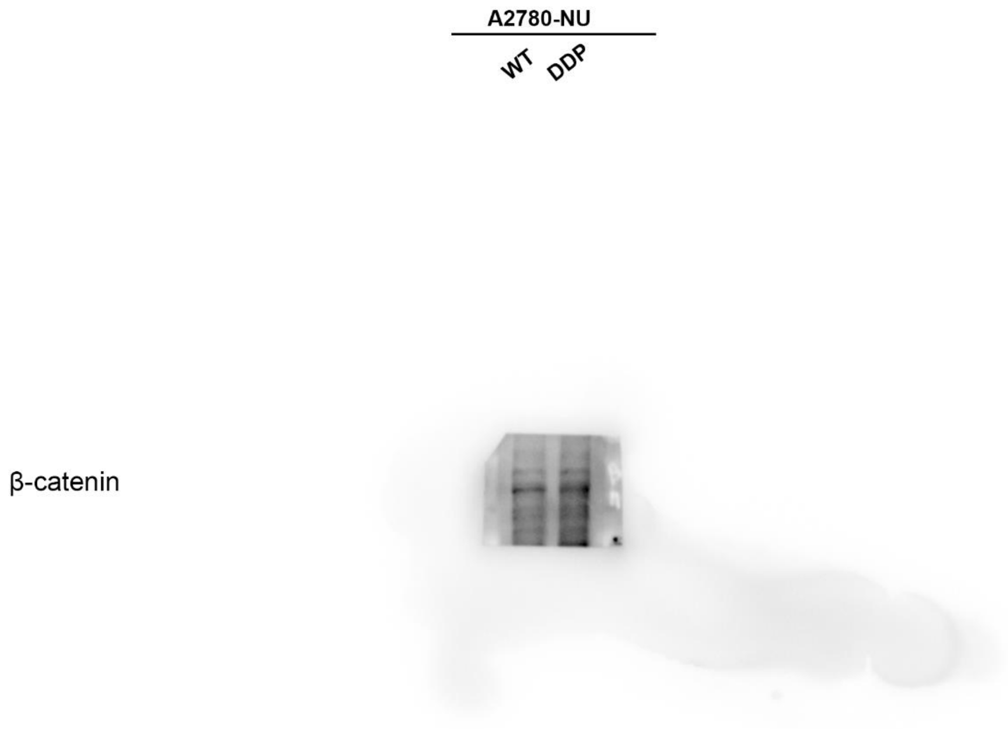

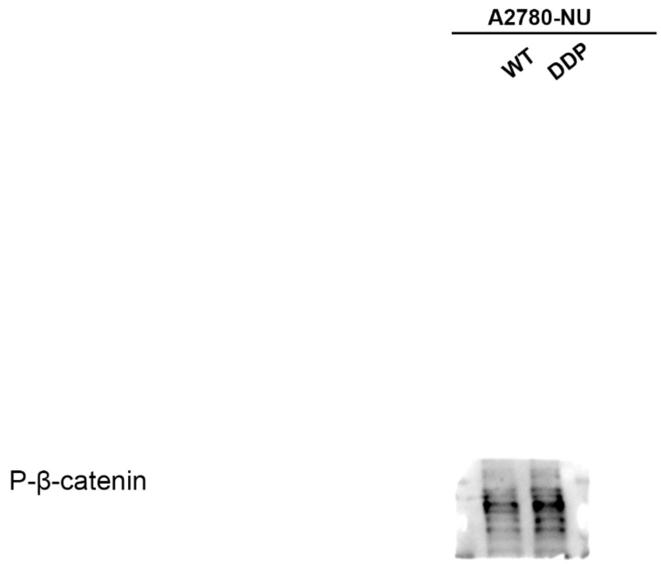
A2780-NU
WT
DDP
P-β-catenin

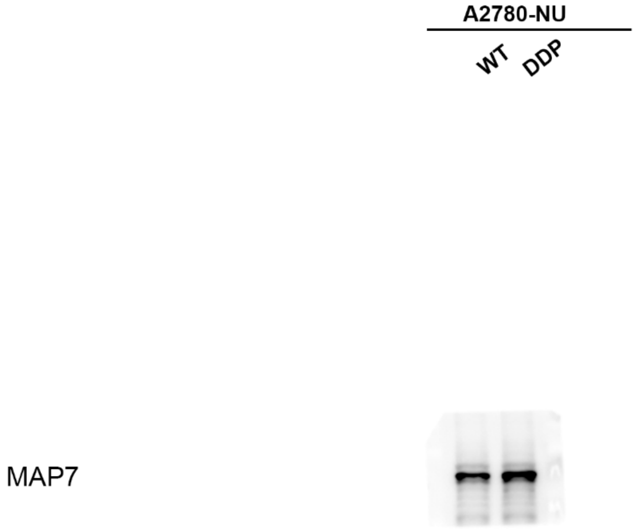
A2780-NU
WT
DDP
MAP7

A2780-NU

WT DDP

GAPDH

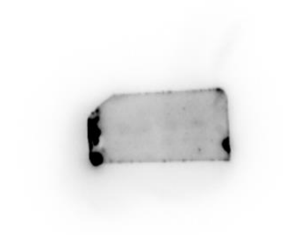

A2780-NU

WT DDP

H3

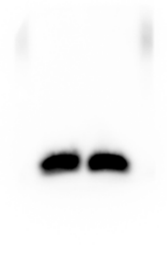

Figure 6D-INPUT

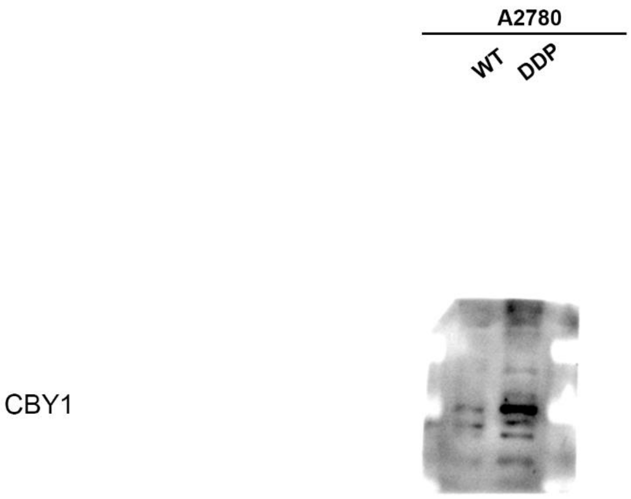

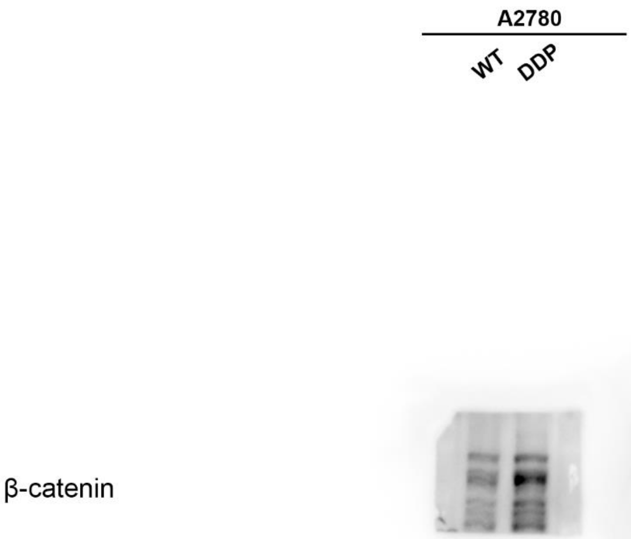

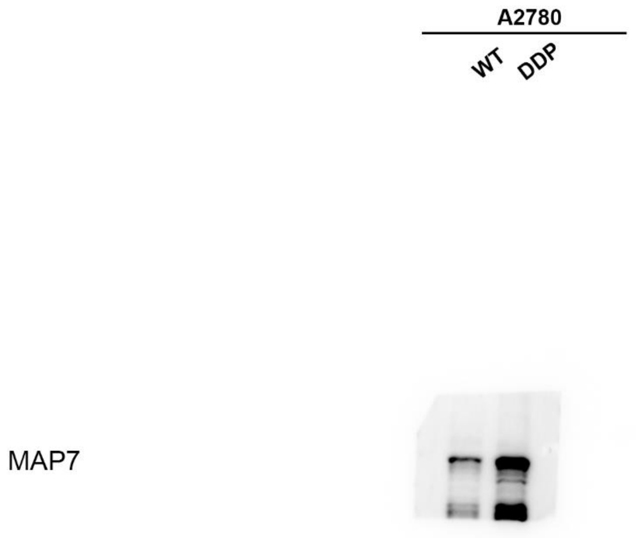
A2780
WT
DDP
MAP7

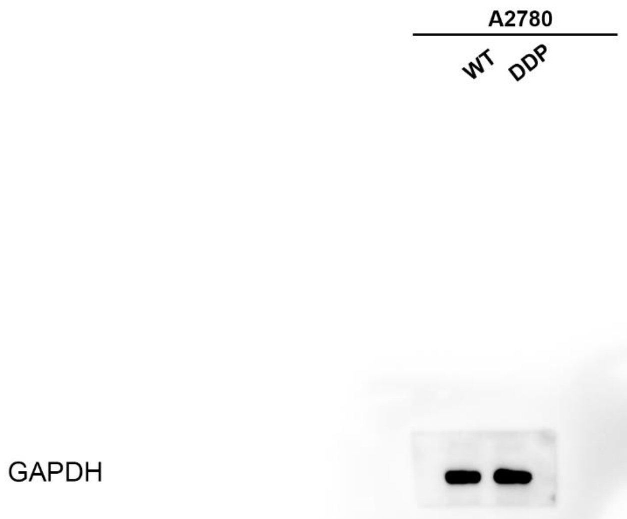
A2780
WT
DDP
GAPDH

Figure 6D-L

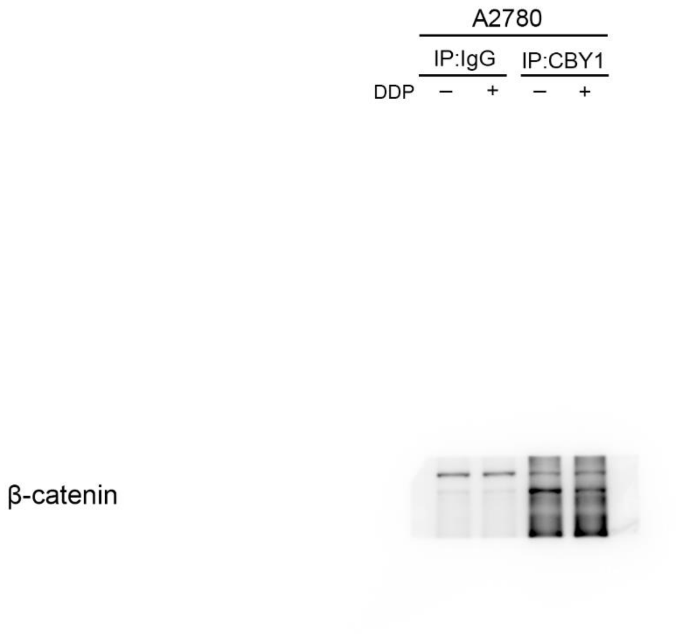
A2780
IP:IgG
IP:CBY1
DDP
–
+
–
+
β-catenin

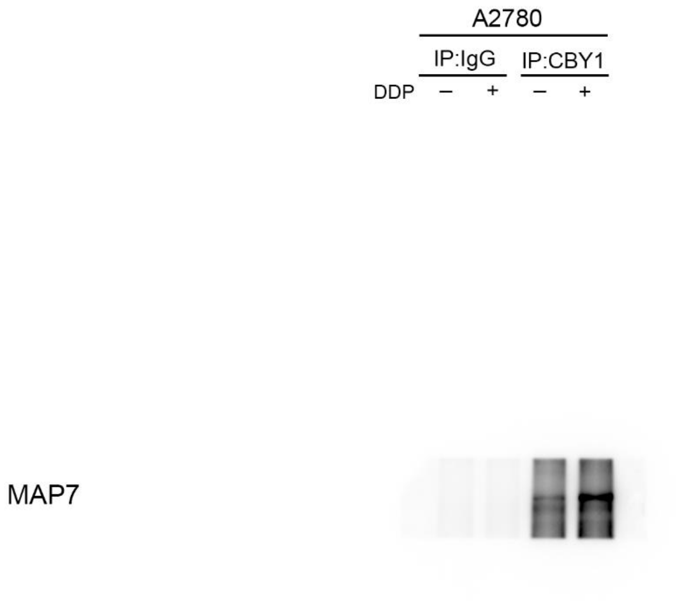
A2780
IP:IgG
IP:CBY1
DDP
–
+
–
+
MAP7

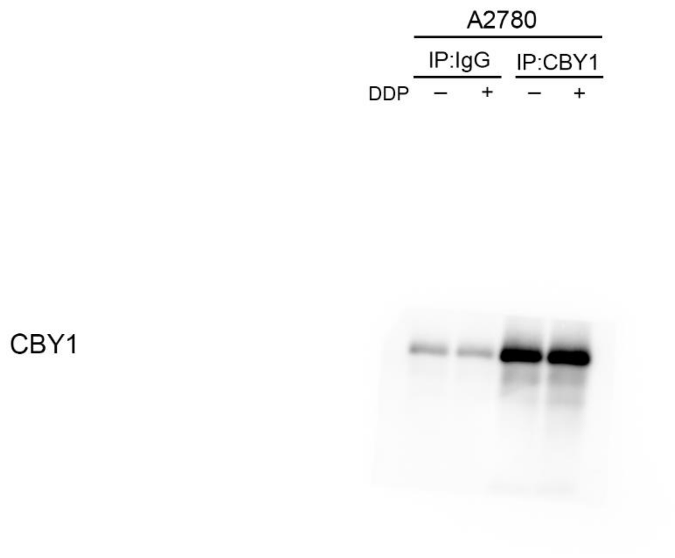
A2780
IP:IgG
IP:CBY1
DDP
–
+
–
+
CBY1

Figure 6D-M

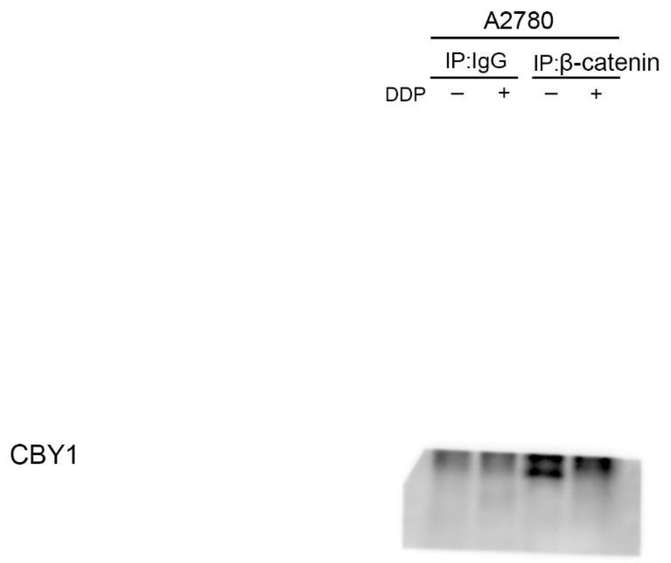
A2780
IP:IgG
IP:β-catenin
DDP
–
+
–
+
CBY1

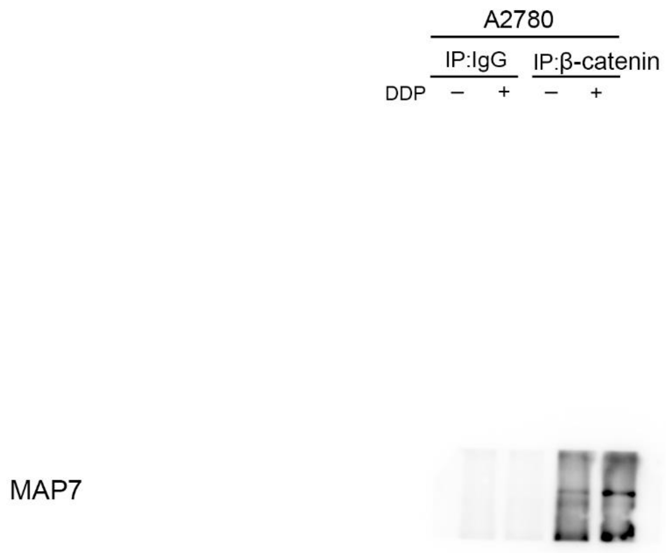
A2780
IP:IgG
IP:β-catenin
DDP – + – +
MAP7

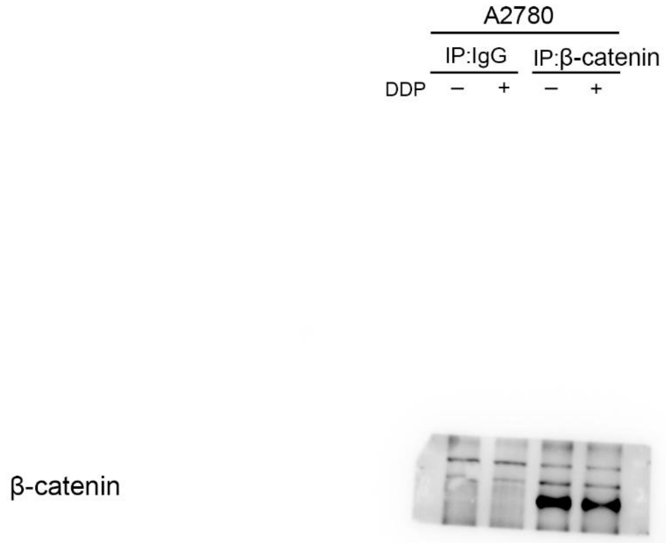
A2780
IP:IgG
IP:β-catenin
DDP – + – +
β-catenin

Figure 6D-R

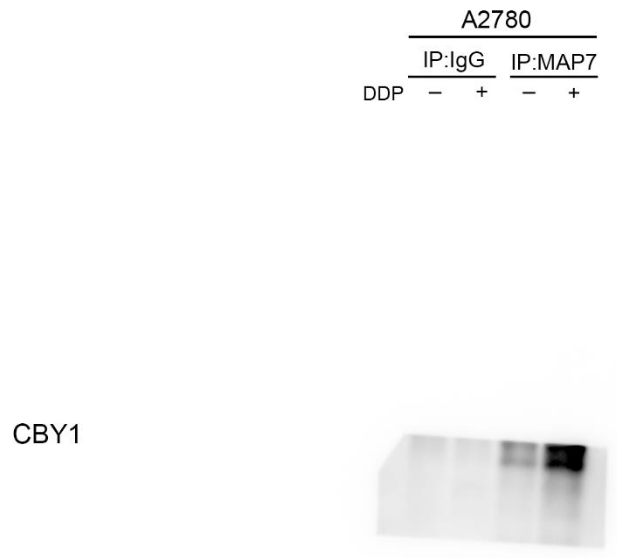
A2780
IP:IgG
IP:MAP7
DDP
–
+
–
+
CBY1

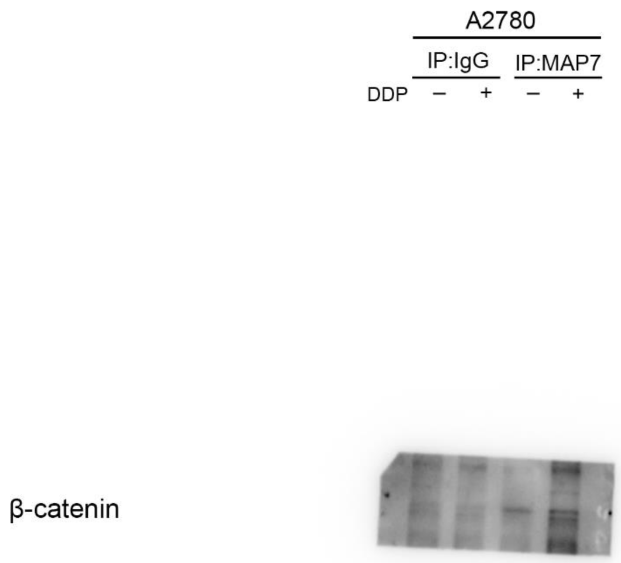
A2780
IP:IgG
IP:MAP7
DDP
–
+
–
+
β-catenin

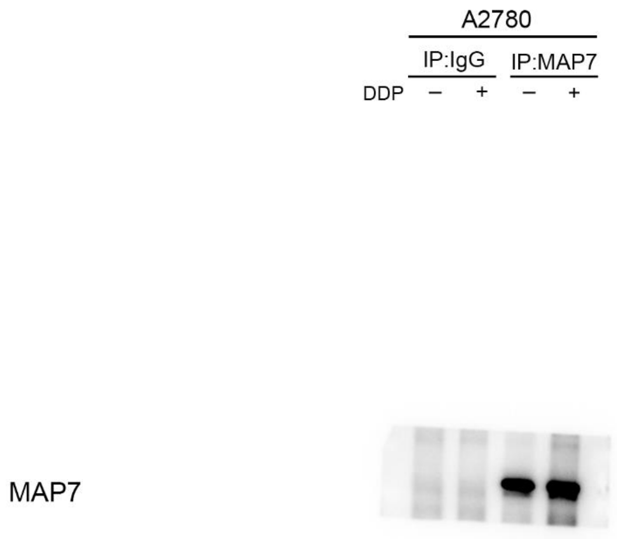
A2780
IP:IgG
IP:MAP7
DDP
–
+
–
+
MAP7
